# The Sall2 transcription factor promotes cell migration regulating focal adhesion turnover and integrin β1 expression

**DOI:** 10.3389/fcell.2022.1031262

**Published:** 2022-11-09

**Authors:** Elizabeth Riffo, Mario Palma, Matías I. Hepp, Diego Benítez-Riquelme, Vicente A. Torres, Ariel F. Castro, Roxana Pincheira

**Affiliations:** ^1^ Laboratorio de Transducción de Señales y Cáncer, Departamento de Bioquímica y Biología Molecular, Facultad de Ciencias Biológicas, Universidad de Concepción, Concepción, Chile; ^2^ Laboratorio de Investigación en Ciencias Biomédicas, Departamento de Ciencias Básicas y Morfología, Facultad de Medicina, Universidad Católica de la Santísima Concepción, Concepción, Chile; ^3^ Millennium Institute on Immunology and Immunotherapy, ICOD, Facultad de Odontología, Universidad de Chile, Santiago, Chile

**Keywords:** Sall2, cell migration, focal adhesion (FA), focal adhesion kinase (FAK), integrin β1, mouse embrionic fibroblasts (MEFs)

## Abstract

SALL2/Sall2 is a transcription factor associated with development, neuronal differentiation, and cancer. Interestingly, *SALL2/Sall2* deficiency leads to failure of the optic fissure closure and neurite outgrowth, suggesting a positive role for SALL2/Sall2 in cell migration. However, in some cancer cells, *SALL2* deficiency is associated with increased cell migration. To further investigate the role of Sall2 in the cell migration process, we used immortalized *Sall2* knockout (*Sall2* ^*−/−*^) and *Sall2* wild-type (*Sall2* ^*+/+*^) mouse embryonic fibroblasts (iMEFs). Our results indicated that Sall2 positively regulates cell migration, promoting cell detachment and focal adhesions turnover. *Sall2* deficiency decreased cell motility and altered focal adhesion dynamics. Accordingly, restoring Sall2 expression in the *Sall2* ^*−/−*^ iMEFs by using a doxycycline-inducible Tet-On system recovered cell migratory capabilities and focal adhesion dynamics. In addition, Sall2 promoted the autophosphorylation of Focal Adhesion Kinase (FAK) at Y397 and increased integrin β1 mRNA and its protein expression at the cell surface. We demonstrated that SALL2 increases *ITGB1* promoter activity and binds to conserved SALL2-binding sites at the proximal region of the *ITGB1* promoter, validated by ChIP experiments. Furthermore, the overexpression of integrin β1 or its blockade generates a cell migration phenotype similar to that of *Sall2* ^*+/+*^ or *Sall2* ^*−/−*^ cells, respectively. Altogether, our data showed that Sall2 promotes cell migration by modulating focal adhesion dynamics, and this phenotype is associated with SALL2/Sall2-transcriptional regulation of integrin β1 expression and FAK autophosphorylation. Since deregulation of cell migration promotes congenital abnormalities, tumor formation, and spread to other tissues, our findings suggest that the SALL2/Sall2-integrin β1 axis could be relevant for those processes.

## Introduction

SALL2/Sall2 is a transcription factor member of the Spalt-like (SALL) family conserved in many organisms, from nematodes to humans. SALL2 has an N-terminal zinc finger domain of the C2HC type, a glutamine-rich region, and several double and triple zinc fingers of the C2H2 type throughout its structure (Sweetman and Münsterberg, 2006; De Celis and Barrio, 2009). The SALL2 gene contains two alternative promoters, P1 and P2 controlling the expression of two main isoforms, E1 and E1A, respectively (Ma et al., 2001). These isoforms differ in the first 25 amino acids at the N-terminal domain. The E1 isoform contains a nuclear localization sequence, and a conserved repressor motif that is not present in E1A (Lauberth and Rauchman, 2006), suggesting isoform-specific functions. The E1 isoform is restricted to specific tissues, such as the brain, kidney, thymus, testis, and colon, while E1A has ubiquitous expression (Ma et al., 2001; Hermosilla et al., 2017). SALL2 was associated with development, neuronal differentiation, and cancer (Hermosilla et al., 2017; Álvarez et al., 2021). SALL2 deregulates in various cancer types, but its role in the disease is not entirely understood. Studies using mouse embryonic fibroblasts (MEFs) derived from the Sall2 knockout model suggest that SALL2/Sall2 behaves as a tumor suppressor by negatively regulating cell cycle progression (Sato et al., 2003; Hermosilla et al., 2018) and by inducing cellular apoptosis during genotoxic stress (Escobar et al., 2015). Consistent with these findings, SALL2 is downregulated in ovarian, lung, and colon cancer (Li et al., 2004; Chai et al., 2011; Farkas et al., 2021). However, clinical reports show that SALL2 is upregulated in other cancer types, such as glioblastoma and synovial sarcoma, suggesting an oncogenic role (Li et al., 2002; Nielsen et al., 2003; Estilo et al., 2009; Suvà et al., 2014).

Like other members of the SALL family, SALL1 and SALL4 (Wolf et al., 2014; Yuan et al., 2016; Itou et al., 2017), SALL2 was associated with the cell migration process. SALL2 silencing promoted cell proliferation, migration, and invasion in A2780 ovarian cancer cells (Miao et al., 2017). In addition, overexpression of SALL2 in radioresistant esophageal squamous cell carcinoma (ESCC) decreased cell migration and chemosensitivity (Luo et al., 2017). These studies suggest that SALL2 inhibits cell migration in these cancer contexts. Conversely, SALL2/Sall2 deficiency was associated with coloboma, a congenital disability characterized by failure to close the optic fissure during the embryonic development of the eye, causing blindness (Kelberman and Schlaepfer, 2017). Additionally, SALL2 interacts with the neurotrophin receptor p75NTR. It is required for nerve growth factor (NGF)-dependent neurite outgrowth of pheochromocytoma of the rat adrenal medulla (PC12) cells and hippocampal primary neurons (Pincheira et al., 2009). Because cell movement is needed for both closure of the optic fissure and neurite outgrowth, they suggest a positive role for SALL2/Sall2 in the migratory process in a normal context.

Cell migration is a complex and highly regulated process relevant to embryonic development, wound healing, and tumor dissemination. It initiates with cell polarity, followed by the generation of membrane protrusions and focal adhesions (FAs) at the leading edge, disassembly of FAs at the cell rear, and then cell traction (Trepat et al., 2012; SenGupta et al., 2021). For cells to migrate, they must sense different environmental signals through adhesion receptors, namely integrins, forming specialized FAs complexes, which mediate responses to these cues (Huttenlocher and Horwitz, 2011; Conway and Jacquemet, 2019). Integrins are a family of heterodimeric cell surface receptors consisting of one α and one β subunit. There are 18 α-subunits and 8 β-subunits, which can combine into 24 different heterodimers that recognize overlapping but distinct sets of extracellular ligands (Humphries et al., 2006). Integrin binding specificity is determined by their extracellular domain that recognizes diverse matrix ligands, including fibronectin (e.g., α5β1, α4β1), collagen (e.g., α1β1, α2β1), and laminin (e.g., α2β1, α6β1). In leukocytes, integrins also bind to soluble ligands such as the complement component iC3b and other cells by binding to ICAMs (αLβ2, αMβ2) and VCAM-1 (α4β1) molecules (Huttenlocher and Horwitz, 2011; Fagerholm et al., 2019). After integrin ligand-binding, several adaptor proteins like talin, vinculin, and paxillin, bind to the integrin cluster and recruit proteins with tyrosine kinase activity, such as focal adhesion kinase (FAK) (Wehrle-Haller, 2012a; Marsico et al., 2018). Once recruited in the adhesion site, FAK is autophosphorylated at tyrosine 397, generating a chain of successive phosphorylation events that modulate focal adhesion (FA) assembly-disassembly dynamics (Hamadi et al., 2005; Mitra, 2005). Polarized cell migration is characterized by asymmetric adhesion dynamics, where nascent adhesions mature into FAs or disassemble. The FAs disassembly involves weakening or disrupting the integrin-ECM and/or integrin-cytoskeletal linkages, with mechanisms that involve calpain-mediated proteolysis of FA proteins, endocytosis, and recycling of integrins, and degradation of the extracellular matrix (Wozniak et al., 2004; Wehrle-Haller, 2012b). Because of the essential role of integrins in cell migration and invasion, their deregulation leads to severe consequences. The absence of β1 is lethal. The absence or defective functions of β2, which plays essential roles in immune responses and inflammation (Gahmberg et al., 2019; Sun et al., 2021), results in leukocyte adhesion deficiency (LAD) syndromes. Defects in the αIIbβ3 integrin may result in Glanzmann’s thrombasthenia. The absence of leukocyte α4β1, LFA-1, and Mac-1 results in increased leukocytosis and susceptibility to microbial infections (Gahmberg et al., 2019). Integrins’ altered expression patterns and activities are frequent in many cancers, which could further promote tumor metastasis *via* downstream signaling pathways (Hamidi and Ivaska, 2018).

The role of SALL2/Sall2 in cell migration is controversial and mechanistically poorly explored. We used immortalized mouse embryonic fibroblasts (iMEFs) derived from previously characterized Sall2 knockout (KO) mice (Sato et al., 2003; Hermosilla et al., 2018) further to investigate the role of Sall2 in cell migration. Contrary to the previous findings in ovarian and esophageal squamous cell carcinoma (ESCC) cancer cell lines, our studies showed that Sall2 promotes cell migration. Sall2 regulated membrane protrusions, cell detachment, FAs maturation, and disassembly. Mechanistically, Sall2 increased FAK autophosphorylation at Y397. It positively regulated integrin β1 expression, increasing integrin protein levels and its availability at the cell surface. Finally, we demonstrated that Sall2 increases integrin β1 mRNA level by directly binding to and transactivating the *ITGB1* promoter. Thus, our study identified integrin β1 as a novel Sall2 target. Taken together, we show that Sall2 promotes cell migration by regulating FA dynamics and increasing integrin β1 expression.

## Materials and Methods

### Reagents

Nocodazole (NZ; M1404), fibronectin (FN; F0895), protease inhibitor cocktail (P8340), phosphatase inhibitor cocktail II (P5726), polybrene (TR-1003), KAPA SYBR FAST qPCR Master Mix (2X) kit (KK4601) and antibodies raised against SALL2 (HPA004162), α-Tubulin (T902), FLAG (M2, F1804) and γ-Tubulin (T6557) were obtained from Sigma-Aldrich (St. Louis, MO United States). Lipofectamine 2000 (11668019), Alexa Fluor 488, 555 and 643 conjugated secondary antibodies (goat anti-mouse), Alexa Fluor 488 phalloidin (A12379) and the PE-conjugated CD29 antibody (eBioscience, #12-0291-82) were purchased from Invitrogen (Carlsbad, MI, United States). FAK Inhibitor compound PF562,271 was from Laviana pharma corporation (JS, China). Doxycycline (#14422) was from Cayman chemical (Ann Arbor, MI, United States). Puromycin dihydrochloride (sc-108071), normal mouse IgG2A (sc-3878), Integrin β1 (M-106, sc-8978), Integrin β1 (A-4, sc-374429), Integrin α4 (B-2, sc-376334), Integrin α6 (F-6, sc-374057), Calpain 2 (E-10, sc-373966), FAK (H-1, sc-1688), PTEN (A2B1, sc-7974), Talin (C-9, sc-365875), Integrin β3 (N-20, sc-6627) and Vinculin (7F9, sc-73614) antibodies were obtained from Santa Cruz Biotechnology (Dallas, TX, United States). Phospho-FAK Tyr397 (#3283) was purchased from Cell Signaling Technology (Danvers, MA, United States). Histone H3 (Ab1791) antibody was obtained from Abcam (Cambridge, United Kingdom). Acetyl-Histone H4 (06-598) antibody was purchased from Millipore (Burlington, MA, United States). Horseradish peroxidase–conjugated secondary antibodies (donkey anti-goat; goat anti-rabbit and goat anti-mouse) and Hoechst 33342 were obtained from Bio-Rad (Hercules, CA, United States).

### Plasmids

The SV40 large T antigen expression pBSSVD2005 was a gift from David Ron (Addgene plasmid # 21826; http://n2t.net/addgene:21826; RRID: Addgene_21826). The pCW57-MCS1-2A-MCS2 (Addgene plasmid # 71782; http://n2t.net/addgene:71782; RRID: Addgene_71782) and pCW57-MCS1-P2A-MCS2 (Hygro) (Addgene plasmid # 80922; http://n2t.net/addgene:80922; RRID:Addgene_80922) were a gift from Adam Karpf, (Barger et al., 2019). For lentiviral infection, the pCMV-dR8.2 dvpr (Addgene plasmid # 8455; http://n2t.net/addgene:8455; RRID: Addgene_8455) and pCMV-VSV-G (Addgene plasmid # 8454; http://n2t.net/addgene:8454; RRID: Addgene_8454) were a gift from Bob Weinberg (Stewart et al., 2003). The EFIa-iTGB1 plasmid was a gift from Joan Massague (Addgene plasmid # 115799; http://n2t.net/addgene:115799; RRID:Addgene_115799) (Er et al., 2018). The pCDNA.3 FLAG_SALL2 was previously generated using the pCMV2-FLAG_SALL2 plasmid (Pincheira et al., 2009). To generate pCW57 Tet-On FLAG mSall2_E1A plasmid, FLAG mSall2_E1A was PCR amplified from pCMV2(NH)-FLAG mSall2_E1A, previously generated using the full-length mouse Sall2 coding sequence synthetized by GeneScrip (Escobar et al., 2015), using the following oligonucleotides: forward, 5′-GCA​GGT​CGA​CAT​GGA​CTA​CAA​AGA​CGA​TGA​C -3′ and reverse, 5′-GCA​GCC​TAG​GTC​ATG​GCA​TGG​TGG​GGT​CAT​CTT​T -3′ and then was subcloned into pCW57-MCS1-2A-MCS2 using SalI and AvrII restriction sites. The *ITGB1* promoter_pGL4.30 was kindly provided by Dr. Masakazu Toi (Kyoto University, Japan; MTA (2019) to RP, Universidad de Concepción, Chile).

### Isolation of primary MEFs and genotyping

*Sall2* KO mice (Sato et al., 2003) were obtained by collaboration with Dr. Ruichi Nishinakamura (Kumamoto University, Japan; MTA to RP, Universidad de Concepción, Chile). *Sall2* ^*+/+*^ and *Sall2* ^*−/−*^ mouse embryonic fibroblasts (MEFs) were isolated and genotyped as previously described (Escobar et al., 2015).

### MEFs immortalization and lentiviral transduction

Primary *Sall2* ^*+/+*^ and *Sall2* ^*−/−*^ MEFs (passage 4) were immortalized using simian virus 40 (SV40) large T antigen based on a modified protocol from Zhu et al. (Zhu et al., 1991). For transfection, we used Lipofectamine 2000 and 2 μg SV40 large T antigen expression vector. After cell transfection, we proceeded to select for low density. To complete the immortalization process, 5-6 post-transfection passages were carried out. The inducible reconstitution of Sall2 in immortalized *Sall2* ^*−/−*^ MEFs, was produced by transient transfection of pCMV-dR8.2 dvpr, pCMV-VSV-G, and pCW57 Tet-On FLAG mSall2_E1A into HEK293T cells in a 10 cm dish with Lipofectamine 2000 reagent according to the manufacturer’s instructions. Viral supernatants were collected at 24 h and passed through a 0.2 μm filter. *Sall2* ^*−/−*^ iMEFs were infected with viral supernatants containing polybrene at 8 μg/ml. For positive clone selection, a fresh medium containing 5 μg/ml of puromycin was used for 72 h. For Sall2_E1A induction, iMEFs were treated with doxycycline (1,000 ng/ml) for 24–48 h before each experiment. Like the generation of the inducible mSall2_E1A cellular model, the integrin β1 overexpression in *Sall2* ^*−/−*^ iMEFs was performed by transfecting HEK293T cells with the EFIa-iTGB1 plasmid or empty vector (negative control). For positive clone selection, a fresh medium containing 400 μg/ml of hygromycin was used for 96 h. After selection, the generation of the stable models was evaluated through western blot.

### Cell culture

*Sall2* ^*+/+*^ and *Sall2* ^*−/−*^ iMEFs, Tet-On Sall2 iMEFs, HEK293T (ATCC, Manassas, VA, United States; CRL-3216) and a previously generated *SALL2* KO HEK293 cell model (Hermosilla et al., 2018) were cultured in Dulbecco’s modified Eagle’s medium (DMEM; Corning) supplemented with 10% (v/v) fetal bovine serum (FBS; Hyclone), 1% glutamine (Invitrogen) and 1% penicillin/streptomycin (Invitrogen). The cell lines used in this study were regularly tested (6 months) for *mycoplasma* using EZ-PCR *Mycoplasma* Test Kit (Biological Industries).

### Cell viability

iMEFs were seeded at 1 × 10^5^ cells per 12-well plate. After 24 h, cells were serum-starved for 16 h. Survival rate was determined by using the trypan blue exclusion method. All experiments were performed in triplicate.

### Wound healing assay

Cell culture dishes were previously covered with fibronectin (FN) 2 μg/ml, iMEFs were seeded at 4 × 10^4^ cells per 24-well plate. After 48 h, cells were serum-starved overnight and wounded with a pipette tip. Phase contrast images were acquired at 0 and 16 h. Wound closure percentage was calculated by the change in wound area between 0 and 16 h. All experiments were performed in quintuplicate. For the integrin β1 blocking study, *Sall2* ^*+/+*^ iMEFs were incubated at the time of wounding with 20 mg/L of anti-integrin β1 antibody (CD29) or non-immune IgG (mouse IgG2A). Non-immune IgG was used as a negative control. IncuCyte S3 system was used to perform a time-lapse video of the wound closure by iMEFs, every 30 min for a total period of 19 ^**½**^ h.

### Transwell migration assay

Transwell inserts (Costar, 6.5-mm diameter, 8 um pore size) were previously covered with FN (2 μg/ml). iMEFs (2 × 10^4^) were seeded into the upper chamber in serum-free medium, complete medium was placed into the bottom chamber. After 4 h, the cells remaining at the upper membrane surface were removed and migrating cells (on the lower membrane surface) were fixed with 10% v/v acetic acid, 10% v/v methanol solution, and stained with 0.4% (w/v) crystal violet for 10 min at room temperature. All experiments were performed in triplicate.

### Immunofluorescence microscopy

iMEFs (4 × 10^4^) were seeded on coverslips previously covered with FN (2 μg/ml). After 48 h, a wound was performed as an activator of migration. After wound completion (4 or 16 h), cells were fixed with 4% paraformaldehyde, permeabilized with 0.1% Triton X-100 and incubated overnight at 4°C with primary antibodies (in blocking buffer, 3% bovine serum albumin in phosphate-buffered saline). After washing, fixed cells were incubated with Hoechst 33342 and Alexa fluor-conjugated secondary antibodies for 2 h. Images were obtained with LMS 780 spectral confocal system (Zeiss). For cell polarity analysis, the microtubule-organizing center (MTOC) was detected using γ-tubulin antibody. To measure the protrusion formation, actin filaments were stained with Alexa Fluor 488 phalloidin. For FAs analysis, vinculin protein was detected using a specific antibody. Identical exposure times and zoom (40x) were used for comparisons and quantification.

### Cell detachment assay

iMEFs were seeded at 1 × 10^5^ cells per 12-well plate. After 24 h, cells were incubated in ethylenediaminetetraacetic acid (EDTA) 0.05 mM at 37°C for 5, 10, and 15 min, respectively. As a control, untreated cells were used. Cells were fixed and stained with crystal violet for 10 min at room temperature. Then, crystal violet was extracted in 10% (v/v) acetic acid, and the absorbance at 590 nm was measured. The adherent cells were expressed as percentages, where 100% is A_590_, corresponding to the number of adherent cells before EDTA-detachment, and 0% is A_590_ of an empty well (no cells attached). All experiments were performed in triplicate.

### Cell spreading

iMEFs (4 × 10^5^) in suspension were allowed to attach and spread onto FN-coated 60-mm dishes (2 μg/ml) for the indicated periods (0, 10, 15, and 30 min). Samples were prepared for western blot analysis.

### Focal adhesion dynamics analysis

For focal adhesion synchronization, iMEFs (2 × 10^4^) were seeded on coverslips previously covered with FN (2 μg/ml). After 24 h, cells were treated with 10 μM nocodazole (NZ) in serum-free medium for 2 ^**½**^ h. NZ was washed-out with serum-free medium at 5, 10, 15, and 30 min. Subsequently, cells were fixed and prepared for immunofluorescence and stained with anti-vinculin antibody (Alexa 488). For inhibition of FAK autophosphorylation, PF562,271 was used at 1 μM concentration in conjunction with NZ treatment. The number of focal adhesions per cell was quantified at all time points. Identical exposure times and zoom (63x) were used for comparisons and quantification.

### Western blot analysis

Proteins from cell lysates (30-50 μg of total protein) were fractionated by sodium dodecyl sulfate-polyacrylamide gel electrophoresis and transferred for 16 h at 30 V to polyvinylidene fluoride (PVDF) membrane (Millipore; IPVH00010) using a wet transfer system. The PVDF membranes were blocked for 1 h at room temperature in 5% nonfat milk in TBS-T (TBS with 0.1% Tween) and incubated with primary antibody at an appropriate dilution at 4°C overnight in blocking buffer. After washing, the membranes were incubated for 1 h at room temperature with horseradish peroxidase-conjugated secondary antibodies diluted in TBS-T buffer. Immunolabeled proteins were visualized by ECL (RPN2209) or ECL prime (RPN2232) (Cytiva, Marlborough, MA, United States). For protein expression *in vivo*, proteins were extracted from brain tissues of 6–8 weeks old *Sall2* ^*+/+*^ and *Sall2* ^*−/−*^ mice as previously described (Hermosilla et al., 2018).

### Flow cytometry

For integrin β1 expression at the membrane surface, 2 × 10^5^ iMEFs were washed, detached in 0.25% trypsin, and collected in 200 μL of cold sorting buffer (2% bovine serum albumin in phosphate-buffered saline). Then, the cells were incubated with 1 μg of PE-conjugated CD29 antibody in the dark for 30 min at 4°C. After incubation, cells were washed, resuspended in 500 μL of sorting buffer, and sorted using a BD FACSAria III cell sorter (BD Biosciences).

### Bioinformatic analysis

Identification of putative SALL2/Sall2 sites was performed using a previously reported binding site matrix (consensus sequence GGG (T/C)GGG) (Gu et al., 2011) in Transcriptional Regulatory Element Database (TRED) (http://rulai.cshl.edu/cgi-bin/TRED/tred.cgi?process=analysisMatrixForm) with a cutoff score = 7.5 (Jiang et al., 2007). Sequences analyzed [–2000 bp from transcription start site (+1)] were obtained from Eukaryotic Promoter Database (EPD) (http://epd.vital-it.ch/). The comparative analysis of *ITGB1* promoter derived from ChIP-seq data was performed as previously described (Farkas et al., 2021).

### Real-time quantitative reverse transcription-PCR

Total RNA was extracted from cells with TRIzol reagent (Invitrogen; 15596026) according to the manufacturer’s instructions. RNA was treated with Turbo Dnase (Invitrogen; AM1907) to eliminate any residual DNA from the preparation. RNA (1 μg) was reverse transcribed using the Maloney murine leukemia virus reverse transcriptase (Invitrogen; 28025-01) and 0.25 μg of Anchored Oligo (dT) 20 Primer (Invitrogen; 12577-011). Real-time PCR was performed using KAPA SYBR FAST qPCR Master Mix (2X) kit and the AriaMX Real-Time PCR System (Agilent, Santa Clara, CA, United States) according to the manufacturer’s instructions. The thermal cycling variables used were as follows: 40 cycles at 95°C for 5 s and 60°C for 20 s. To control specificity of the amplified product, a melting-curve analysis was carried out. No amplification of unspecific product was observed. Amplification of RNA polymerase II (*Polr2*) was carried out for each sample as an endogenous control. Primers used for real-time reactions are summarized in Supplementary Table S1. The relative expression ratio of the *Itgb1* gene was calculated using the standard curve method, using untreated *Sall2* ^*+/+*^ iMEFs as reference.

### Transient transfections and reporter gene assays

To evaluate *ITGB1* promoter transcriptional activity, *SALL2* KO HEK293 cells were transiently co-transfected with 1 μg of *ITGB1* promoter, 0.125 μg of RSV-β-galactosidase (β-Gal), and 2 μg of FLAG_SALL2 (pCDNA.3 FLAG SALL2) or 1 μg of vector control per well. After 24 h, the transfected cells were lysed with reporter lysis 5X buffer (E4030; Promega, Madison, WI, United States) and centrifugated at 14000 × g to pellet cell debris. The supernatant was then assayed for luciferase and β-Gal activity using the manufacturer’s suggested protocols (Promega; E1483 and E4740, respectively). Luminescent reporter activity was measured using a Luminometer (Victor3; Perkin- Elmer). All transfections were normalized to β-Gal activity and performed in triplicate. Luciferase values were expressed as fold induction relative to the pGL3 vector control.

### Chromatin immunoprecipitation assay

*SALL2* KO HEK293 cells were seeded at 1 × 10^6^ cells per 100-mm dish. After 24 h, cells were transiently transfected with 2 μg of pCDNA.3 FLAG_SALL2. Cell nuclei were sonicated in 300 μL of sonication buffer, using a Bioruptor plus sonicator (Diagenode) (40 times, 15 s on/15 s off each time, high potency), obtaining DNA lengths between 300 and 600 bp. Immunoprecipitations were carried out overnight at 4°C using 2 μg of FLAG (anti-FLAG antibody), 1 μg of H3 (anti-histone H3; Abcam), or 1 μg of acH4 (anti-histone H4 acetylated; Millipore) and 25 μg of chromatin. After crosslinking reversion, DNA was purified using DNA Clean & Concentrator™-25 kit (D4033, Zymo Research, Irvine, CA, United States). DNA was analyzed by PCR directed to *ITGB1* promoter SALL2-specific proximal I and proximal II regions (-251/-127) and (-631/-297). The *ITGB1* promoter region (-1986/-1878) was used as a negative control for SALL2 binding. Primers used for qPCR reactions are summarized in Supplementary Table S2. All PCR reactions were performed using KAPA SYBR FAST qPCR Master Mix (2X) kit containing 1 μL of input and 4 μL of IP samples.

### Image quantification and statistical analysis

Confocal immunofluorescence and phase contrast images were manually analyzed using the ImageJ software. Data were analyzed using unpaired *t*-test performed with GraphPad Prism 9.3.1 software. Values averaged from at least three independent experiments were compared. A *p*-value of <0.05 was considered significant.

## Results

### Sall2 promotes cell migration

**FIGURE 1:**
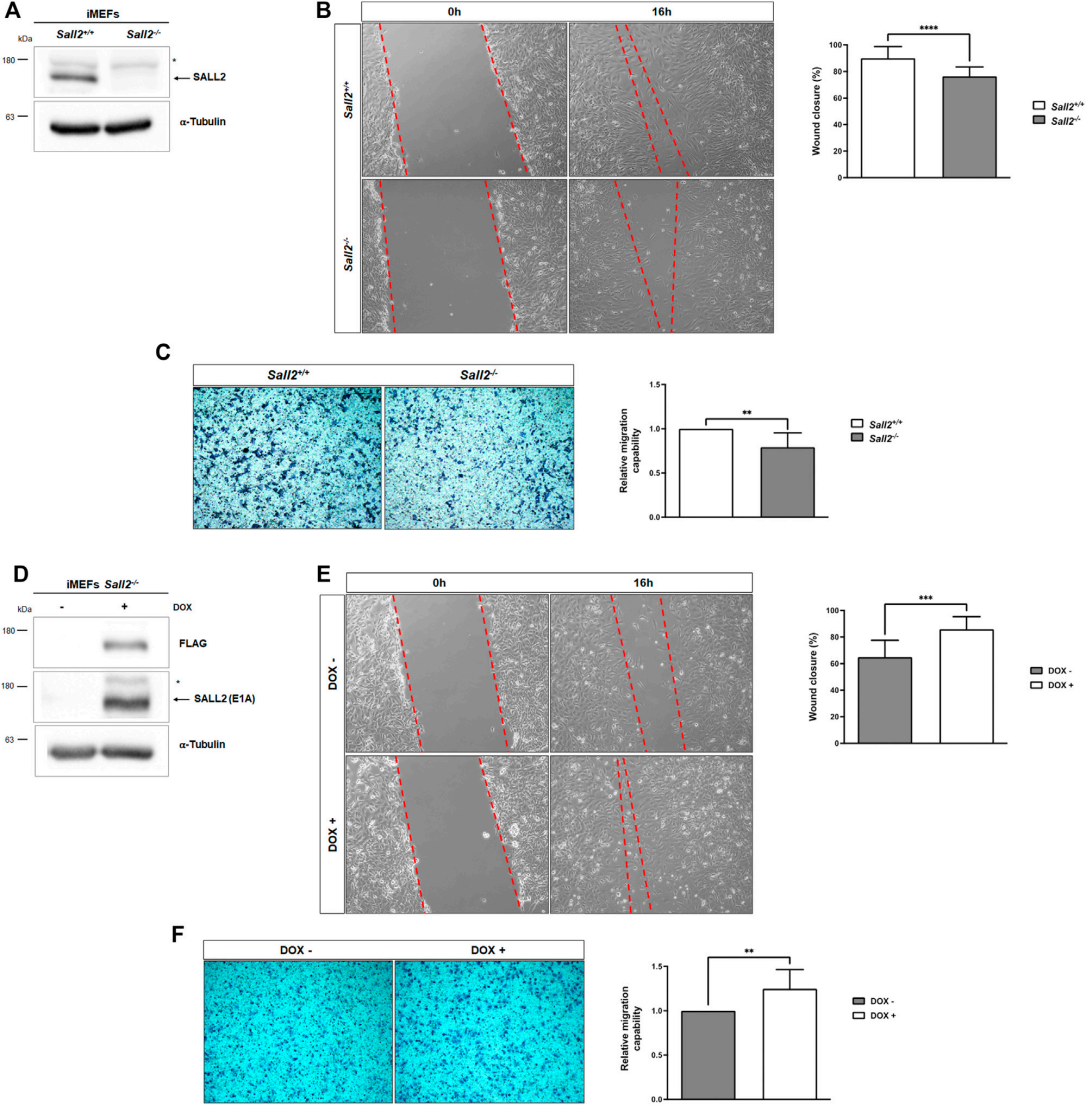
Sall2 promotes iMEFs migration. **(A)** Western blot analysis of endogenous Sall2 in *Sall2* ^*+/+*^ and *Sall2* ^*−/−*^ iMEFs. The arrow indicates Sall2, and the asterisk corresponds to a nonspecific band. α-tubulin was used as the loading control. **(B)** Left, representative phase-contrast images (10x) at 0 and 16 h of *in vitro* wounding from *Sall2* ^*+/+*^ and *Sall2* ^*−/−*^ iMEFs. Right, quantification of wound closure (as a percentage) at 16 h from images obtained in **(B)**. **(C)** Left, representative microscopy images (10x) of transwell inserts from *Sall2* ^*+/+*^ and *Sall2* ^*−/−*^ iMEFs after 4 h of cell migration. Right, quantification of the number of migrated cells from images obtained in **(C)**. **(D)** Western blot analysis of mSall2 E1A isoform (FLAG) expression in the Tet-On Sall2 iMEFs inducible model (doxycycline (1,000 ng/ml, 48 h). **(E)** Left, representative phase-contrast images (10x) at 0 and 16 h of *in vitro* wounding from the Tet-On Sall2 iMEFs. Right, quantification of wound closure (as a percentage) at 16 h from images obtained in **(E)**. **(F)** Left, representative microscopy images (10x) of transwell inserts from the Tet-On Sall2 iMEFs model after 4 h of cell migration. Right, quantification of the number of migrated cells from images obtained in **(F)**. Data is expressed as mean ± SD from three independent experiments performed in quintuplicate and triplicated, respectively (**p = 0.001 to 0.01, ***p = 0.0001 to 0.001, ****p ≤ 0.0001; unpaired *t*-test).

We investigated the role of Sall2 in cell migration by comparing the migration of iMEFs isolated from isogenic *Sall2* knockout (*Sall2* ^*−/−*^) and *Sall2* wild-type (*Sall2* ^*+/+*^) embryos immortalized with the large T antigen (Hermosilla et al., 2018). Figure 1A shows the expression of Sall2 in the *Sall2* ^*+/+*^ iMEFs. We subjected iMEFs to an *in vitro* wound healing assay and evaluated cell migration 16 h post-scratching. As shown in Figure 1B and Supplementary movies S1, S2, *Sall2* ^*−/−*^ iMEFs exhibited a lower percentage of wound closure than the *Sall2* ^*+/+*^ iMEFs (about 76% and 90%, respectively). There were no differences in cell viability between both genotypes at the time and in the conditions of the wound closure analysis (Supplementary Figure S1).

We performed transwell migration assays further to analyze the effects of Sall2 in cell migration. As expected, *Sall2* ^*−/−*^ iMEFs exhibited fewer migratory cells than *Sall2* ^*+/+*^ cells (Figure 1C) (about 21% less). To confirm the role of Sall2 in cell migration, we restored its expression in the *Sall2* ^*−/−*^ iMEFs. Since *Sall2* ^*+/+*^ iMEFs mainly express the Sall2 E1A isoform (Hermosilla et al., 2018), we used a Tet-On system to generate a Sall2 E1A inducible cellular model (Tet-On Sall2 iMEFs) (Figure 1D). Consistent with the above results, induction of Sall2 expression by doxycycline increased cell migration (wound closure percentage from 65% to 86%, Figure 1E) and increased the number of migratory cells (about 20% more) (Figure 1F). Altogether, these results demonstrated that Sall2 promotes cell migration in iMEFs cells.

### Sall2 decreases the lamellipodia size and promotes cell detachment

To determine how Sall2 promotes cell migration, we evaluated the requirement of Sall2 expression on the different steps involved in cellular movement, including cell polarity, membrane protrusion formation, and cell adhesion.

First, we assessed cell polarization. Like other studies in MEFs, we evaluated polarity establishment at 4 h after wounding (Grande-García et al., 2007; Tomar et al., 2009). The maintenance of cell polarity was evaluated at 16 h (Li and Gundersen, 2008), when the wound edges were still distinguishable, and migration was almost complete (Supplementary Figures S2B,E). Cell polarity was measured by analyzing the localization of the microtubule-organizing center (MTOC) relative to the nucleus following the induction of cell migration. The MTOC positioning was grouped into four grades, as previously described (Silverman-Gavrila et al., 2011), where grade 1 corresponds to a highly polarized state, grade 2 polarized, grade 3 non-polarized, and grade 4 to a highly non-polarized state (Supplementary Figure S2A). Our results showed no significant differences between Sall2 genotypes in cell polarization at 4 and 16 h (Supplementary Figures S2C,F). Similar results were observed when comparing highly polarized grade (G1) with medium polarized grade (G2). As shown in Supplementary Figures S2D,G, there were no significant differences between *Sall2* ^*−/−*^ and *Sall2* ^*+/+*^ cells. All these results indicated that Sall2 is not required to induce cell polarity, polarization grade, and cell polarity maintenance over time.

**FIGURE 2:**
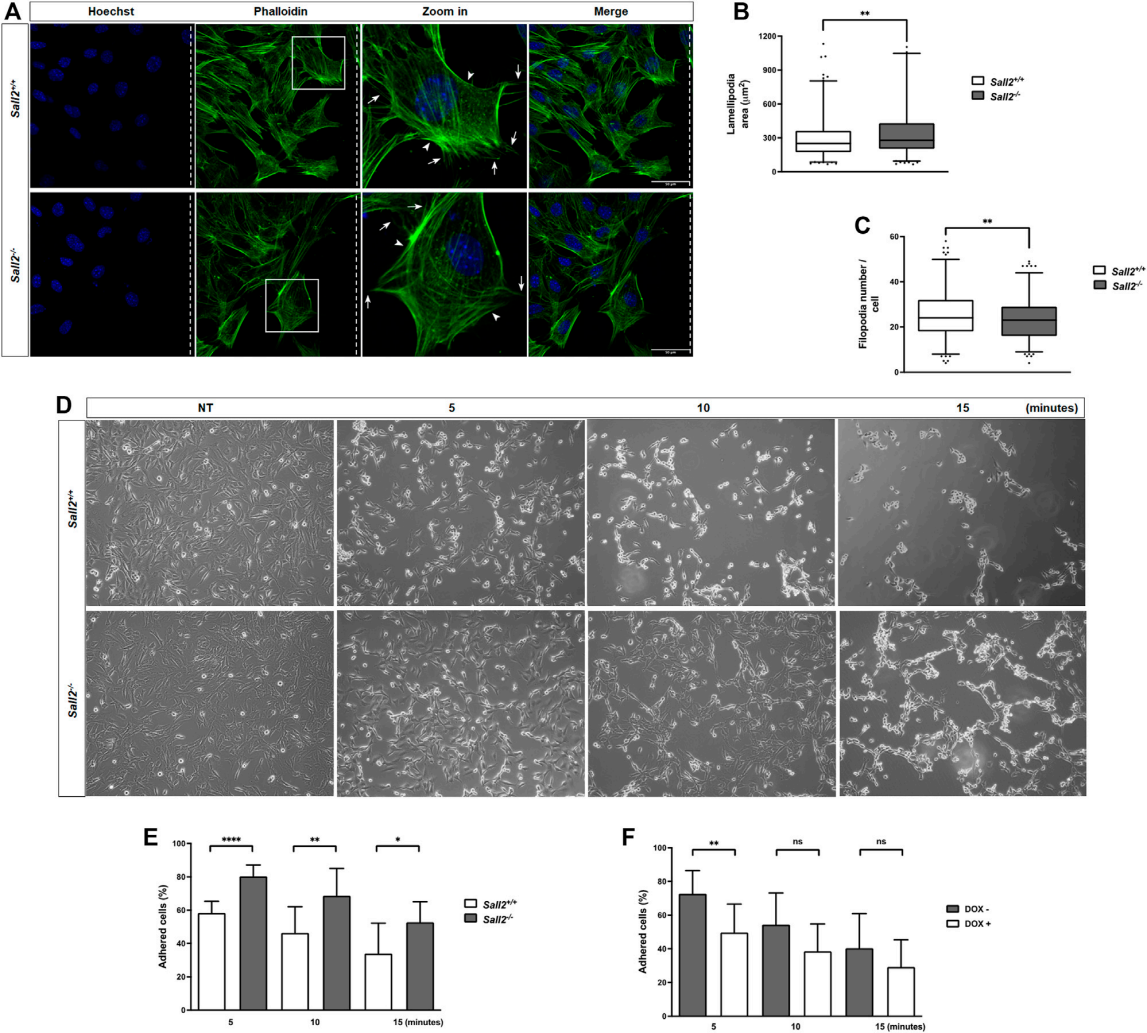
Sall2 decreases the lamellipodia size and promotes cell detachment. **(A)** Representative confocal images (40 ×) from *Sall2* ^*+/+*^ and *Sall2* ^*−/−*^ iMEFs after cell migration induction at 16 h. Cells were fixed and stained with phalloidin (F- actin, green) and Hoechst (nuclei, blue). Dashed lines indicate the position of the wound, and arrows and arrowheads show the filopodia and lamellipodia, respectively. **(B)** Quantification of lamellipodia area from images obtained in **(A)**. **(C)** Quantification of filopodia number from images obtained in **(A)**. Data of cell protrusions analysis are shown as a box-and-whiskers plot from three independent experiments. The whiskers represent the 2.5th and 97.5th percentile, the box extends from the 25th to the 75th percentile and the line indicates the median. For each experiment at least 100 cells were analyzed per genotype. **(D)** *Sall2* ^*+/+*^ and *Sall2* ^*−/−*^ iMEFs were treated with EDTA-containing solution for the indicated times, then fixed and evaluated by microscopy. Representative phase contrast images (10x) from each time point are shown. **(E,F)** Quantification of the percentage of adherent cells from *Sall2* ^*+/+*^ and *Sall2* ^*−/−*^ iMEFs **(E)** and the Tet-On Sall2 iMEFs inducible model **(F)** performed as described in the MATERIALS AND METHODS section. Data of cell detachment assays are expressed as mean ± SD from three independent experiments performed in triplicate. (n.s, not significant, *p = 0.01 to 0.05, **p = 0.001 to 0.01, ****p ≤ 0.0001; unpaired *t*-test).

We next evaluated whether Sall2 affects the membrane protrusions. We stained actin filaments after cell migration induction (Figure 2A) and analyzed the formation of lamellipodium and filopodium structures by quantifying their number, size, and polarization state. As shown in Figures 2B,C, *Sall2* ^*−/−*^ iMEFs depicted a higher lamellipodia area (mean = 352 *versus* 301 mm^2^) and exhibited a slightly lower filopodia number (mean = 23 *versus* 25) than *Sall2* ^*+/+*^ cells. Still, there were no significant differences in the lamellipodia number, lamellipodia polarization state, filipodia length, and filipodia polarization state when comparing genotypes (Supplementary Figures S3A–D). Our data suggest that Sall2 mainly regulates the area of the lamellipodia.

Additionally, we evaluated whether Sall2 regulates the cell adhesion process. We assessed cells binding to the extracellular matrix by comparing the detachment rate induced by adding EDTA. As shown in Figure 2D, iMEFs treated with an EDTA-containing solution resulted in a time-dependent detachment of cells. Interestingly, *Sall2* ^*−/−*^ cells exhibited a higher percentage of adherent cells (80% at 5 min, 69% at 10 min, and 53% at 15 min) than *Sall2* ^*+/+*^ iMEFs (58% at 5 min, 46% at 10 min and 34% at 15 min) (Figure 2E). Accordingly, induction of Sall2 expression in the Tet-On Sall2 iMEFs significantly decreased the percentage of adherent cells at all times of EDTA treatment (47% at 5 min, 38% at 10 min, and 29% at 15 min), as observed in *Sall2* ^*+/+*^ cells (Figure 2F). Our results support that Sall2 promotes cell detachment, suggesting a novel role of Sall2 in cell adhesion.

### Sall2 decreases focal adhesions maturation and promotes focal adhesion dynamics

**FIGURE 3:**
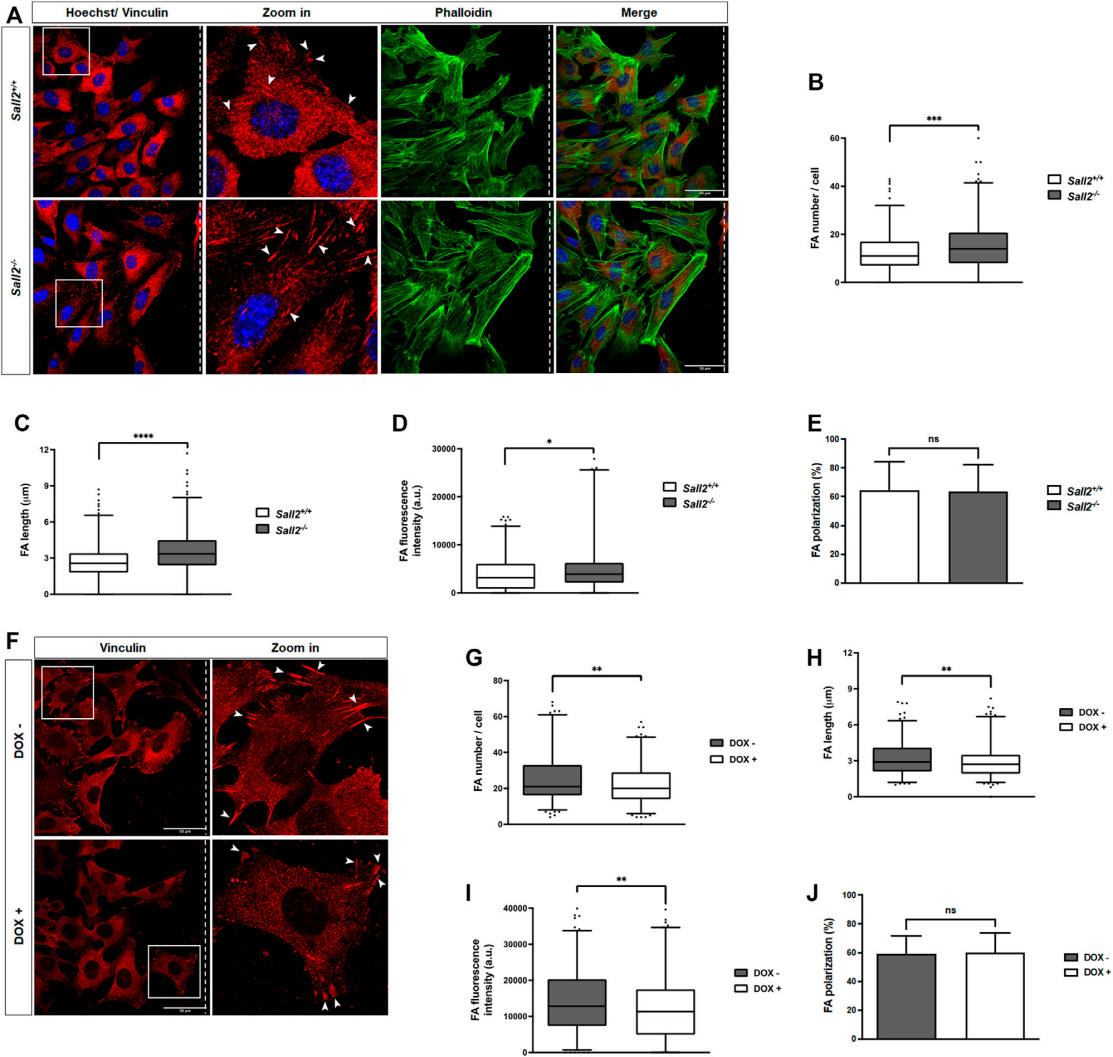
Sall2 decreases focal adhesions maturation. **(A)** Representative confocal images from *Sall2* ^*+/+*^ and *Sall2* ^*−/−*^ iMEFs after cell migration induction at 16 h (40x). Cells were fixed, and FAs were stained with anti-vinculin antibody (red), phalloidin stained F- actin (green), and Hoechst stained the nuclei (blue). Dashed lines indicate the position of the wound, and the arrowheads show FA. **(B–E)** Quantification of FA number per cell **(B)**, FA length **(C)**, FA fluorescence intensity **(D)**, and FA polarization **(E)** from *Sall2* ^*+/+*^ and *Sall2* ^*−/−*^ cells. **(F)** Representative confocal images from the Sall2 Tet-On iMEFs inducible model after cell migration induction at 16 h (40x). **(G–J)** Quantification of FA number per cell **(G)**, FA length **(H)**, FA fluorescence intensity **(I)**, and FA polarization **(J)** from the Tet-On Sall2 iMEFs. For each experiment at least 100 cells were analyzed per genotype. Data are shown as a box-and-whiskers plot from three independent experiments. The whiskers represent the 2.5th and 97.5th percentile, the box extends from the 25th to the 75th percentile, and the line indicates the median. (n.s, not significant, *p = 0.01 to 0.05, **p = 0.001 to 0.01, ***p = 0.0001 to 0.001, ****p ≤ 0.0001; unpaired *t*-test).

To further investigate the role of Sall2 in cell adhesion, we evaluated whether Sall2 regulates focal adhesions by quantifying their number, length, and fluorescence intensity. After induction of cell migration in *Sall2* ^*+/+*^ and *Sall2* ^*−/−*^ iMEFs and the Tet-On Sall2 iMEFs model, we measured focal adhesion (FA) by immunocytochemistry of vinculin (Figures 3A,F), a protein involved in FA formation (Legate and Fässler, 2009; Wehrle-Haller, 2012a). As shown in Figure 3B, the FA number in *Sall2* ^*−/−*^ iMEFs was significantly higher than in *Sall2* ^*+/+*^ cells (a mean of 15 *versus* 12 FA/cell). In addition, FA length (mean = 3.5 *versus* 2.7 mm) and FA fluorescence intensity (mean = 5,034 *versus* 4,088 a.u.) in *Sall2* ^*−/−*^ iMEFs were also significantly higher in comparison with *Sall2* ^*+/+*^ cells (Figures 3C,D). Accordingly, Sall2 induction by doxycycline in Tet-On Sall2 iMEFs decreased the number, length, and fluorescence intensity of FAs, like the *Sall2* ^*+/+*^ cells (Figures 3G–I). No significant differences in the polarization state of FAs between *Sall2* ^*−/−*^ and *Sall2* ^*+/+*^ cells (Figure 3E) or after induction of Sall2 expression (Figure 3J) were observed. Since FA formation is a dynamic process where the FAs can disassemble or mature into stabilized complexes characterized by increased size (Gardel et al., 2010; Wehrle-Haller, 2012b), our results suggested that Sall2 negatively regulates the maturation of focal adhesions, as well as their number.

**FIGURE 4:**
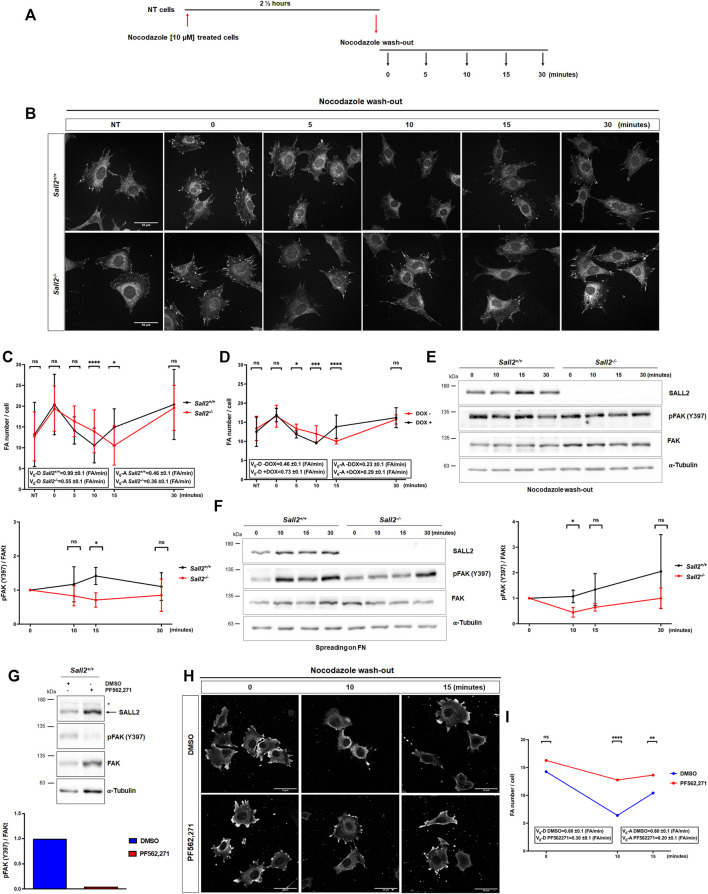
Sall2 promotes focal adhesion dynamics. **(A)** Schematic representation of nocodazole treatment and wash-out. **(B)** Representative confocal images (63x) from *Sall2* ^*+/+*^ and *Sall2* ^*−/−*^ iMEFs incubated with 10 μM of nocodazole in serum-free medium. Nocodazole was washed-out at different times. Cells were fixed, and FAs were stained with anti-vinculin antibody (white). **(C,D)** Quantification of FA number per cell from *Sall2* ^*+/+*^ and *Sall2* ^*−/−*^ iMEFs **(C)** and the Tet-On Sall2 iMEFs inducible model **(D)**. Graphs included initial velocity (Vo-D) of FA disassembly and initial velocity (Vo-A) of FA assembly. For each experiment, at least 60 cells were analyzed per condition. **(E)** Left, FAK autophosphorylation (Y397) was evaluated by western blot at different times of nocodazole wash-out. Right, FAK autophosphorylation was quantified by densitometric analysis with respect to t = 0 min of nocodazole wash-out and normalized to total FAK and α-tubulin expression. **(F)** Left, FAK autophosphorylation (Y397) was evaluated by western blot at different times of spreading on fibronectin (FN). Right, FAK autophosphorylation was quantified by densitometric analysis with respect to t = 0 min of cell spreading and normalized to total FAK and α-tubulin expression. **(G)** Western blot of FAK autophosphorylation (Y397) and densitometric quantification from *Sall2* ^*+/+*^ iMEFs treated with DMSO or 1 μM PF562,271. **(H)** Representative confocal images from *Sall2* ^*+/+*^ iMEFs at different times of nocodazole wash-out treated with DMSO or 1 μM PF562,271. FAs were stained with anti-vinculin antibody (white). **(I)** Quantification of FA number per cell from *Sall2* ^*+/+*^ iMEFs treated with DMSO or 1 μM PF562,271. Initial velocity (Vo-D) of FA disassembly and initial velocity (Vo-A) of FA assembly are indicated. For each experiment, at least 60 cells were analyzed per condition. Data are expressed as mean ± SD from three independent experiments (n.s, not significant, *p = 0.01 to 0.05, **p = 0.001 to 0.01, ***p = 0.0001 to 0.001, ****p ≤ 0.0001; unpaired *t*-test).

Considering these observations, we next evaluated whether Sall2 regulates FA dynamics. FAs were stabilized by microtubule depolymerization using nocodazole (Mendoza et al., 2013) at the concentration and time previously optimized (Figure 4A; Supplementary Figure S4). As expected, nocodazole increased the number of mature FAs compared with the non-treated cells ‘NT’. In contrast, nocodazole wash-out was followed by a progressive decrease of FA number, as observed in both genotypes (Figure 4B). Interestingly, *Sall2* ^*−/−*^ iMEFs exhibited delayed kinetics of FA disassembly (about 5 min) compared with *Sall2* ^*+/+*^ cells (Figure 4C). In addition, *Sall2* deficiency decreased the FA disassembly’s initial velocity (v_0_-D from 0.99 ± 0.1 to 0.55 ± 0.1). Conversely, we did not observe significant differences in FA assembly (kinetics and initial velocity of FA assembly, v0-A) when comparing *Sall2* genotypes (Figure 4C). Accordingly, the induction of Sall2 expression significantly accelerated FAs disassembly (v_0_-D from 0.46 ± 0.1 to 0.73 ± 0.1), but not their assembly (Figure 4D). These results were supported by biochemical analysis of FAK autophosphorylation at Y397 (Hamadi et al., 2005). Phospho-Y397-FAK was evaluated after nocodazole release and during spreading on fibronectin at different time points (both approaches are widely used to assess changes in FAK activation, Nader et al., 2016; Erusappan et al., 2019). Phospho-Y397-FAK levels after 15 min of nocodazole wash-out were significantly lower in *Sall2* ^*−/−*^ iMEFs than in control cells (Figure 4E **)**. Similarly, the phosphorylation of FAK at Y397 after 10 min of cell spreading was significantly lower in *Sall2* ^*−/−*^ iMEFs than in *Sall2* ^*+/+*^ cells (Figure 4F). We further investigated the role of FAK activity in the Sall2-dependent regulation of FA dynamics. To this aim, *Sall2* ^*+/+*^ iMEFs were treated with PF562,271, a specific inhibitor of FAK. As expected, PF562,271 decreased FAK- Y397 phosphorylation (Figure 4G). Like the *Sall2* ^*−/−*^ iMEFs phenotype, the treatment with PF562,271 decreased the FAs disassembly of *Sall2* ^*+/+*^ iMEFs after 10 min of nocodazole wash-out. (Figure 4H). We observed a decrease in the initial velocity of FA disassembly (v_0_-D from 0.80 ± 0.1 to 0.30 ± 0.1) and assembly (v_0_-A from 0.80 ± 0.1 to 0.20 ± 0.1) (Figure 4I). Altogether, these results indicate that Sall2 promotes FAK activation and, consequently, the focal adhesion turnover.

### Sall2 promotes integrin β1 expression

**FIGURE 5:**
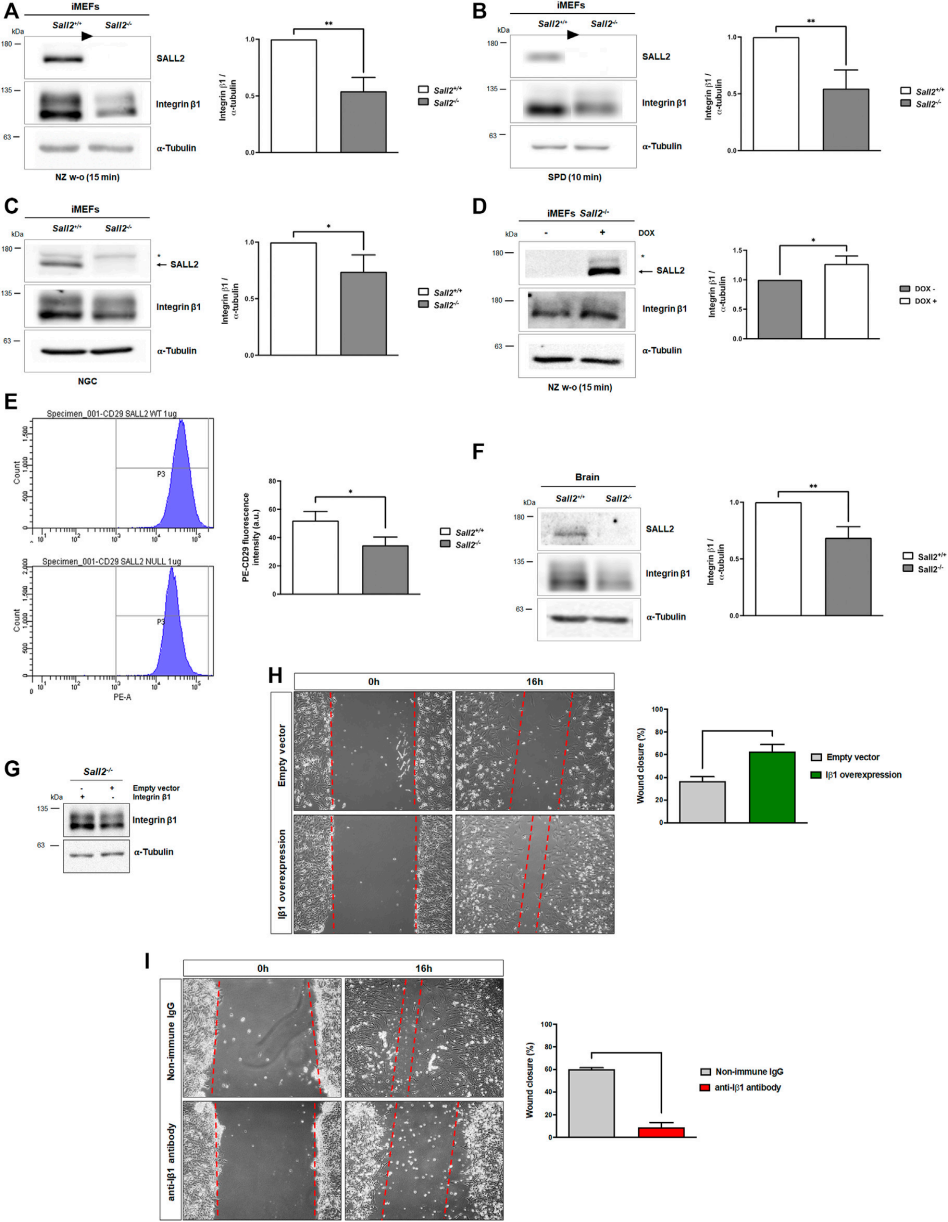
Sall2 promotes integrin β1 expression. **(A–C)** Representative blots of integrin β1 expression, and densitometric quantification, from *Sall2* ^*+/+*^ and *Sall2* ^*−/−*^ iMEFs after 15 min of nocodazole wash-out treatment (NZ w-o) **(A)**, 10 min of cell spreading (SPD) on FN **(B)**, and in normal growth conditions (NGC) **(C)**. **(D)** Representative blot of integrin β1 protein level, and densitometry, from the Tet-On Sall2 iMEFs inducible model after 15 min of NZ w-o. **(E)** Flow cytometry analysis of the cell surface expression of integrin β1 from *Sall2* ^*+/+*^ and *Sall2* ^*−/−*^ iMEFs. **(F)** Left, a representative blot of brain tissues from 6 to 8-week-old *Sall2* ^*+/+*^ and *Sall2* ^*−/−*^ mice. Right, integrin β1 densitometry from brain tissues. The arrow indicates Sall2, and the asterisk corresponds to a nonspecific band. The arrowheads in **(A,B)** indicate cropped unrelated columns and subsequent splicing of the blot. α-tubulin was used as loading control in western blot analysis. **(G)** Western blot analysis of integrin β1 overexpression in *Sall2* ^*−/−*^ iMEFs. **(H)** Left, representative phase-contrast images (10x) at 0 and 16 h of *in vitro* wounding from *Sall2* ^*−/−*^ iMEFs overexpressing integrin β1. Right, quantification of wound closure (as a percentage) at 16 h from images obtained in Left. **(I)** same as in H, but for *Sall2* ^*+/+*^ iMEFs incubated with anti-β1 antibody. Data are expressed as mean ± SD from three independent experiments (*p = 0.01 to 0.05, **p = 0.001 to 0.01;unpaired *t*-test).

To determine the underlying mechanism by which Sall2 promotes FA dynamics, we analyzed the expression of different proteins involved in FA assembly-disassembly, such as integrins, vinculin, talin, and calpain 2. The analysis was performed under the conditions where the highest differences in FAK activation were observed (15 min after nocodazole release and 10 min of cell spreading) and under normal growth conditions. Although *Sall2* ^*+/+*^ cells depicted higher levels of FAK phosphorylation (Figures 4E,F), *Sall2* deficiency was associated with higher FAK protein levels (Supplementary Figures S5B–G). These results suggested that Sall2-mediated FAK activation does not relate to positive regulation of FAK expression. In addition, we found that *Sall2* ^*−/−*^ iMEFs have significantly lower protein levels of integrin β1 than *Sall2* ^*+/+*^ cells in all conditions analyzed (Figures 5A–C). Alpha subunits analysis showed slightly lower integrin α4 levels in *Sall2* ^*−/−*^ iMEFs than *Sall2* ^*+/+*^ cells under normal growth conditions. However, we did not observe significative differences in the integrin α6 expression levels (Supplementary Figure S6). Additionally, we did not observe differences in the expression levels of vinculin, talin, calpain2 (Supplementary Figure S5), and integrin β3 (Supplementary Figure S6).

Considering the functional relevance of β-subunits in the recruitment of several adapters and signaling molecules to form focal adhesions (Schnittert et al., 2018), and since we did not observe changes in the integrin β3 protein levels, we focused our study on integrin β1. We evaluated how Sall2 induction affects integrin β1 levels after 15 min of nocodazole wash-out treatment, where the highest difference in integrin β1 expression was observed. We found that Sall2 expression significantly increases integrin β1 protein level, like the *Sall2* ^*+/+*^ cells (Figure 5D). Additionally, we evaluated the integrin β1 expression at the cell surface of iMEFs by flow cytometry. As expected, *Sall2* ^*−/−*^ iMEFs exhibited a lower integrin β1 expression than *Sall2* ^*+/+*^ cells (mean PE-CD29 fluorescence intensity = 35 *versus* 52 a.u.) (Figure 5E). Previously, we reported detectable Sall2 protein levels in mice tissues, including the brain, cerebellum, and spleen (Hermosilla et al., 2018). To evaluate the significance of the Sall2-dependent regulation of integrin β1 *in vivo*, we compared integrin β1 expression between brain tissues from isogenic *Sall2* ^*+/+*^ and *Sall2* ^*−/−*^ mice. Consistent with our previous results (Figures 5A–D), integrin β1 protein levels were significantly lower in Sall2^*−/−*^ than in *Sall2* ^*+/+*^ tissues (Figure 5F).

To validate that the regulation of integrin β1 expression by Sall2 is directly involved in the cell migration phenotype, we performed functional studies with integrin β1 overexpression or the blocking of integrin β1 function. The integrin β1 overexpression in *Sall2* ^*−/−*^ iMEFs increased cell migration (wound closure percentage from 37% to 63%, Figures 5G,H), similar to that observed in *Sall2* ^*+/+*^ cells. On the other hand, the integrin β1 blockage with an anti β1antibody in *Sall2* ^*+/+*^ iMEFs showed a reduced wound closure percentage (from 60% to 9%, Figure 5I). Together, our results demonstrated that Sall2 positively regulates the integrin β1 expression and promotes cell migration through this regulation.

### Sall2 transcriptionally regulates integrin β1

**FIGURE 6:**
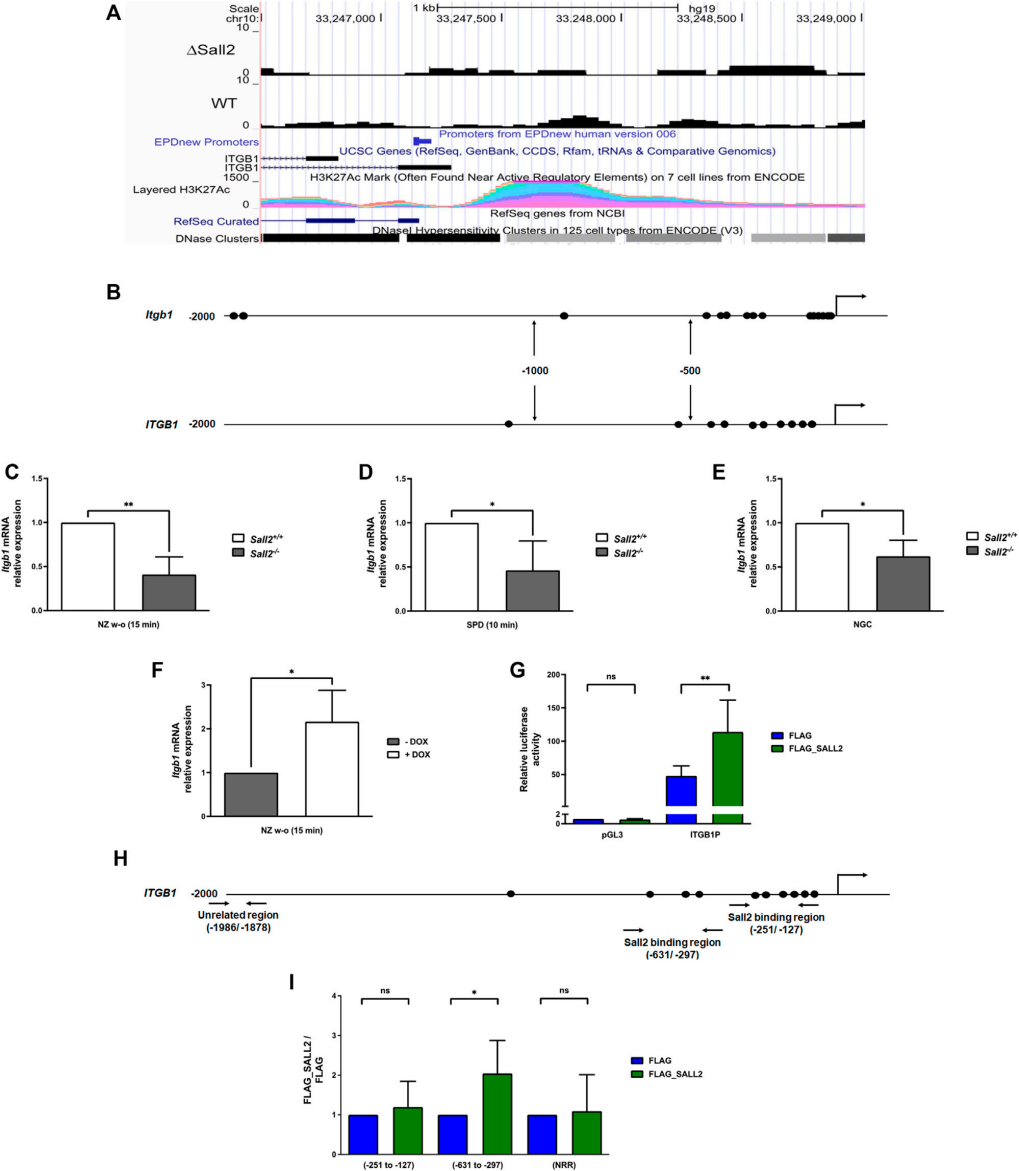
Sall2 transcriptionally regulates integrin β1. **(A)** Bigwig track from *ITGB1* gene, according to reference from NCBI RefSeq genes and UCSC genes (ENCODE), from SALL2 wild-type (WT) and SALL2 knockout HEK293 cells (ΔSall2) ChIP-seq datasets. SALL2 binding enrichment and associated peaks in the promoter region, according with EPDnew Promoters ENCODE data are shown. H3K27Ac enhancer mark and DNase Clusters from ENCODE are also shown, plotted with the UCSC genome browser (https://genome.ucsc.edu/, hg19 track). **(B)** Schematic representation of mouse and human integrin β1 (*Itgb1/ITGB1*) promoters. The putative SALL2/Sall2 binding sites are represented by black ovals. The transcription start site (+1) is represented by arrows. **(C–E)** Integrin β1 mRNA level from *Sall2* ^*+/+*^ and *Sall2* ^*−/−*^ iMEFs were determined by real-time PCR after 15 min of nocodazole wash-out treatment (NZ w-o) **(C)**, 10 min of spreading (SPD) on FN **(D)** and under normal growth conditions (NGC) **(E)**. **(F)** Integrin β1 mRNA levels from the Tet-On Sall2 iMEFs inducible model after 15 min of NZ w-o. RNA polymerase II was used as a normalizer. **(G)** *ITGB1* promoter activity in the absence (FLAG vector) and presence of SALL2 (FLAG_SALL2) was performed as described in the MATERIALS AND METHODS. Luciferase activity was measured from cell lysates and normalized to β-galactosidase activity, and promoter activity was expressed as relative luciferase units (R.L.U). pGL3 vector served as control. **(H)** Schematic representation of human *ITGB1* promoter. Horizontal arrows indicate the location of primers used for qPCR in site-specific ChIP assays. **(I)** Chromatin from *SALL2* KO HEK293 cells transfected with FLAG_SALL2 was immunoprecipitated 24 h after transfection using FLAG antibody. Specific genomic regions of the human *ITGB1* promoter and a nonrelated promoter region (NRR) were analyzed by real-time PCR. Graphs show quantification of the amplified DNA for each immunoprecipitation relative to FLAG. Data are expressed as mean ± SD from three independent experiments (n.s, not significant, *p = 0.01 to 0.05, **p = 0.001 to 0.01; unpaired *t*-test).

To investigate whether Sall2 regulates integrin β1 expression, we initially analyzed a previously published ChIP-seq (Farkas et al., 2021) and compared the *ITGB1* promoter data between *SALL2* wild-type and *SALL2* knockout HEK293 cells. We identified SALL2-binding enrichment and associated peaks in the *ITGB1* promoter region in the *SALL2* ^*+/+*^ cells (Figure 6A). Additionally, using the Transcriptional Regulatory Element Database (TRED) (Jiang et al., 2007), we identified several putative binding sites in the mouse and human integrin β1 promoter (15 sites in the mouse and 30 sites in the human promoter), mainly located in the proximal promoter region, –500 bp to +1 (Figure 6B). Conservation analysis using BLASTn revealed 79% of identity between both species at the proximal region. Next, we evaluated the integrin β1 mRNA level by real-time PCR in the previous three cellular contexts. Figures 6C–E show that *Sall2* ^*−/−*^ iMEFs exhibited significantly lower integrin b1 mRNA than *Sall2* ^*+/+*^ cells under nocodazole release, spreading on FN, and normal growth conditions. Like the protein analysis, the highest mRNA difference was observed under nocodazole release. Accordingly, induction of Sall2 expression increased the integrin β1 mRNA level after 15 min of nocodazole wash-out treatment (Figure 6F).

To determine SALL2 activity at the *ITGB1* promoter, we used *SALL2* KO HEK293 cells co-transfected with a SALL2 expression plasmid and an *ITGB1* promoter reporter previously described (Itou et al., 2017). SALL2 expression significantly increased the activity of the *ITGB1* promoter (Figure 6G). By chromatin immunoprecipitation (ChIP) assays in *SALL2* KO HEK293 cells, we demonstrated that SALL2 binds to the proximal promoter of *ITGB1*, specifically to the -631/-297 region (Figures 6H,I). In contrast, no binding of SALL2 was observed in a nonrelated promoter region (NRR) and the -251/-127 region of the *ITGB1* promoter. The increment of SALL2 binding was correlated with an increase in histone H4 acetylation, a transcriptional activation marker (Supplementary Figure S7). These results support that integrin β1 is a novel Sall2 transcriptional target gene.

## Discussion

Cell migration is an important event in several physiological processes. Deregulation of cell migration leads to misplaced cells and, consequently, abnormal functions, promoting several diseases such as congenital abnormalities, autoimmune syndromes, and primary tumor dissemination (Horwitz and Webb, 2003). A better understanding of the molecular factors that regulate cell migration could lead to novel therapeutic approaches.

In this study, we elucidated the role of the Sall2 transcription factor in the migration of immortalized mouse embryonic fibroblasts. Our studies demonstrated that Sall2 is required for optimal cell migration. Specifically, we found that Sall2 promotes cell detachment, FAK autophosphorylation, and FA dynamics. In addition, we demonstrated that Sall2 positively regulates integrin β1 expression by directly binding to the *ITGB1* promoter.

Previous evidence is consistence with our findings, suggesting that SALL2/Sall2 promotes cell migration*. SALL2* deficiency leads to optic fissure closure failure, causing blindness (Kelberman et al., 2014). In hippocampal neurons, *SALL2* silencing significantly decreased neurite outgrowth (Pincheira et al., 2009). The stages before optic fissure closure, such as optic vesicle evagination, depend on the effective migration (Rembold et al., 2006). On the other hand, neurons must be highly polarized to extend their axon and dendrites (Li and Gundersen, 2008). Thus, these reported phenotypes associate the loss of SALL2/Sall2 function with a decrease in cell migration, reinforcing a positive role of SALL2/Sall2 in this process. Interestingly, two reports contradict this conclusion, associating *SALL2* deficiency with increased cell migration in cancer. Depleting SALL2 in A2780 ovarian cancer cells increased migration and invasion and was associated with the activation of the PI3K/Akt signaling (Miao et al., 2017). Similarly, epigenetic silencing of SALL2 was associated with increased migration ability. Upregulation of SALL2 in ESCC cells decreased the growth and migration of radioresistant cells, which is directly associated with acquiring more aggressive phenotypes (Luo et al., 2017). Considering that no previous studies show a direct role of SALL2/Sall2 in cell migration in a non-cancer context, our results in iMEFs are the first to demonstrate that Sall2 plays a positive role in promoting cell migration.

The apparent controversy on the role of SALL2/Sall2 in the migration of A2780 cancer (Miao et al., 2017) *versus* MEFs, a non-cancer cell model, might relate to several reasons. The A2780 ovarian cell line is associated with amoeboid migration, characterized by the ability to move and invade independently of proteolysis by conferring increased motility and invasion of cells (Beaufort et al., 2014). These cells harbor mutations in *BRCA2, PIK3CA, PTEN,* and *ARID1A* genes, among others, associated with tumor initiation (Beaufort et al., 2014). In contrast, MEFs are associated with a mesenchymal-like phenotype. Mesenchymal cells move slowly, exhibit multiple protrusions, and depend strongly on integrin-mediated adhesions to the extracellular matrix (Panková et al., 2010; Bear et al., 2014). Here, we used immortalized MEFs, which are widely helpful in assessing migratory and invasive capacities (Birch et al., 2016; Prieto et al., 2020). These cells are a simple genetic model characterized mainly by the inactivation of p53 and Rb proteins (Ali and DeCaprio, 2001). Thus, depending on the cellular context and the presence or absence of specific partners, SALL2/Sall2 could affect its transcriptional activity and cellular functions. For example, SALL2 is a tumor suppressor in ovarian cancers but promotes aggressiveness in the glioma context. SALL2 binds target genes, *BAX*, *p16*, and *c-MYC* in ovarian cancer through the canonical GC-rich motif (Gu et al., 2011). In contrast, it binds to AT-rich motifs related to its interaction with SOX2 protein in glioma, reprogramming the tumor-propagating potential of glioblastoma stem-like cells (Suvà et al., 2014).

SALL2 isoforms might also account for different cellular outcomes. Two SALL2 isoforms, E1 and E1A, have been characterized. They are differentially expressed and only differ at the N-terminal end. The E1 isoform contains a repressor domain relevant to its interaction with the Nucleosome Remodeling Deacetylase (NuRD) complex, which is not present in E1A (Lauberth and Rauchman, 2006). A short E1A SALL2 isoform, lacking DNA binding domain and most Zinc finger motifs, is also deregulated in cancer contexts (Farkas et al., 2021). We previously showed that the E1A is the predominant Sall2 isoform in iMEFs (Hermosilla et al., 2018), suggesting that this isoform is responsible for promoting iMEFs’ cell migration. The expression of a different SALL2 isoform in A2780 and ESCC cancer cells may explain the contradictory results but was not established. Therefore, identifying differential functions of SALL2/Sall2 isoforms may resolve its contradictory role in cell migration between cancer and non-cancer cells.

We demonstrated that SALL2 binds to and activates the *ITGB1* promoter transcriptional activity, identifying *ITGB1* as a novel SALL2 target. Accordingly, our studies showed that Sall2 promotes the increase of mRNA and protein levels of integrin β1, correlating with an increase in its availability at the cell surface. Of physiological relevance, we also showed that Sall2 correlated with integrin β1 expression in mouse brain tissues, suggesting a conserved role of Sall2 in integrin β1 regulation, at least in normal conditions. We demonstrated that the regulation of integrin β1 is one of the mechanisms involved in the Sall2-dependent promotion of cell migration in fibroblasts.

We observed a more marked effect on cell migration with fibronectin as substrate (data not shown). Changes in the expression of extracellular matrix proteins could affect fibroblast cell migration. MEFs synthesize several matrix proteins, including fibronectin, laminin, and type I and IV collagen. Fibronectin is the most expressed (Blancas et al., 2011) and associates with the integrin heterodimers α4β1, α5β1, α8β1, and αvβ1 (Humphries et al., 2006). Despite this, there is no previous evidence of extracellular matrix protein expression in the context of Sall2 gain and loss of function. We observed that Sall2 might also regulate integrin α4 protein levels (Supplementary Figure S6). Whether Sall2 regulates the α4β1 heterodimer requires further investigation.

The integrin β1 on the plasma membrane activates through a combination of inside-out and outside-in mechanisms, leading to integrin clustering, FA maturation, and integrin downstream signaling mechanisms (Kechagia et al., 2019). As part of the process, integrin β1 is crucial for recruiting FAK, inducing its autophosphorylation at Y397 and the consequent events that modulate FA dynamics involved in the migration of cells (Wennerberg et al., 2000). Although Sall2 deficient cells show higher basal FAK levels, they have significantly lower levels of phospho-Y397-FAK than the wild-type cells, suggesting that Sall2 promotes FAK autophosphorylation independently of its effect on protein levels. A Sall2-dependent transcriptional mechanism may explain the higher levels of FAK in the Sall2 deficient cells since several putative Sall2 binding sites were identified in the Ptk2 (FAK) promoter (Supplementary Figure S8). Previous studies have shown that Sall2 transcriptionally represses *c-MYC* (Sung et al., 2012), *CCND1,* and *CCNE1* genes transcription (Hermosilla et al., 2018). Similarly, Sall2 could bind and repress Ptk2 promoter activity and FAK expression.

Phosphorylation of FAK at Y397 creates a motif recognized by various SH2 domain-containing proteins, including SRC-Family Kinases (SFKs), phospholipase Cγ (PLCγ), growth-factor-receptor-bound protein-7 (GRB7), the Shc adaptor protein, and the p85 subunit of phosphatidylinositol 3-kinase (PI3K) (Mitra et al., 2005). The FAK–SRC signaling complex acts to recruit and/or phosphorylate several proteins, such as p130Cas and paxillin (Mitra et al., 2005). Although there is no evidence of Sall2 affecting some of these signaling components, the decrease in the autophosphorylation of FAK in the *Sall2* deficient cells agreed with their FA dynamic phenotype. FAK autophosphorylation is an important event for its regulation by other kinases and, thus, the regulation of FA dynamics (Hamadi et al., 2005). Accordingly, *Sall2* deficiency led to a substantial delay in the kinetics of FA disassembly and, consequently, to a slower initial velocity of FA disassembly. This result is consistent with the significantly higher FA length and fluorescence intensity observed in the *Sall2* ^*−/−*^ than in the *Sall2* ^*+/+*^ iMEFs, suggesting a lower state of FA maturation in the wild-type cells contributing to a more dynamic FA turnover and migratory phenotype (Gardel et al., 2010). On the other hand, *Sall2* deficiency results in increased FA number and cell adhesion, slowing cell movement. Consistent with their more adherent phenotype, *Sall2* ^*−/−*^ iMEFs depicted a larger lamellipodia area because the more extensive the cell membrane, the higher the number of FA and hence augmented cell adhesion (McGrath, 2007; Gauthier et al., 2012).

Interestingly, autophosphorylation and activation of Pyk2/FAK are essential for propagating signals that control neurite formation in PC12 and SH-SY5Y cells (Ivankovic-Dikic et al., 2000). Likewise, Sall2 is required for neurite outgrowth of PC12 cells and rat hippocampal neurons (Pincheira et al., 2009). We observed that Sall2 slightly increased the number of filopodia, an important structure for the directional response of the cells (Blanchoin et al., 2014). Filopodia participate in several cellular processes, including cell migration, neurite outgrowth, and wound healing (Mattila and Lappalainen, 2008). Whether the effect of Sall2 in the filopodia number relates to the role of Sall2 at the neuronal level is unknown. However, FAK not only plays a crucial role in cell migration. It also promotes cell survival, regulates transcription (Kleinschmidt and Schlaepfer, 2017), and activates the PI3K signaling pathway (Chen et al., 1996; Reif et al., 2003; Kim and Gumbiner, 2015). Thus, promoting FAK autophosphorylation by Sall2 becomes relevant and requires further investigation. Sall2’s effect on FAK activity and filopodia number could be common mechanisms for its role in neurite outgrowth and migration of iMEFs.

An additional mechanism explaining the role of Sall2 in cell migration could relate to the regulation of PTEN. It is well-known that PTEN regulates many cellular processes. They include cell polarity and migration through its lipid phosphatase activity, which antagonizes PI3K (phosphoinositide 3-kinase) signaling (Worby and Dixon, 2014). It also can regulate cell motility independently of its lipid phosphatase activity (Yamada and Araki, 2001). PTEN dephosphorylates FAK and inhibits cell migration, spreading, and focal adhesion formation of U-87MG cells (Tamura et al., 1998). Studies in breast cancer cells demonstrate that SALL2 silencing induced the AKT/mTOR pathway activation *via* the downregulation of PTEN. The mechanism involves the positive regulation of PTEN through the direct binding of SALL2 to the canonical GC-rich motif in the *PTEN* promoter (Ye et al., 2019). Contrary to the cancer context, we observed higher levels of PTEN in *Sall2* ^*−/−*^ cells (Supplementary Figure S9), which supports PTEN as an alternative or complementary mechanism in the Sall2-mediated FAK activation. This possibility requires further studies.

Interestingly, SALL4, the oncogenic member of the SALL family, is associated with increased cell migration and invasion in gastric and breast cancer cell lines (Yuan et al., 2016; Itou et al., 2017). SALL4 regulates integrins β1 and α6 expression in basal-like breast cancer cells, a cell type with high migratory properties (Itou et al., 2017). SALL4 mediates FAK activation, FA dynamics, and Rho inhibition to promote migration by a mechanism involving the SALL4/integrin α6β1 network (Itou et al., 2017). Unlike SALL4, SALL2/Sall2-dependent cell migration did not involve integrin a6. SALL4 binds DNA at the AT-Rich motif (Kong et al., 2021), but, as shown here, SALL2 binds to the canonical GC-Rich motif (Gu et al., 2011), suggesting a common regulation of *ITGB1* by SALL proteins *via* differential promoter-binding sites. Studies indicate that the regulation of *ITGB1* by specific SALL proteins might be context specific. For example, in breast cancer cells, SALL2 is downregulated, but SALL4 is upregulated (Alvarez et al., 2021). Thus, SALL4 is expected to have a primary role in *ITGB1* regulation. On the other hand, in the glioma context, SALL2 and SALL4 are upregulated, potentially exerting a synergistic effect on the regulation of integrin β1 (Alvarez et al., 2021). In addition to SALL4 and SALL2, studies in breast cancer lines showed that SALL1 inhibition increases cell migration and correlates with decreased cadherin 1 expression. (Wolf et al., 2014). Our findings further support the role of the SALL family members in cell migration. However, how different contexts affect the involvement of SALL proteins in cell migration and integrins regulation requires further investigation.

**FIGURE 7:**
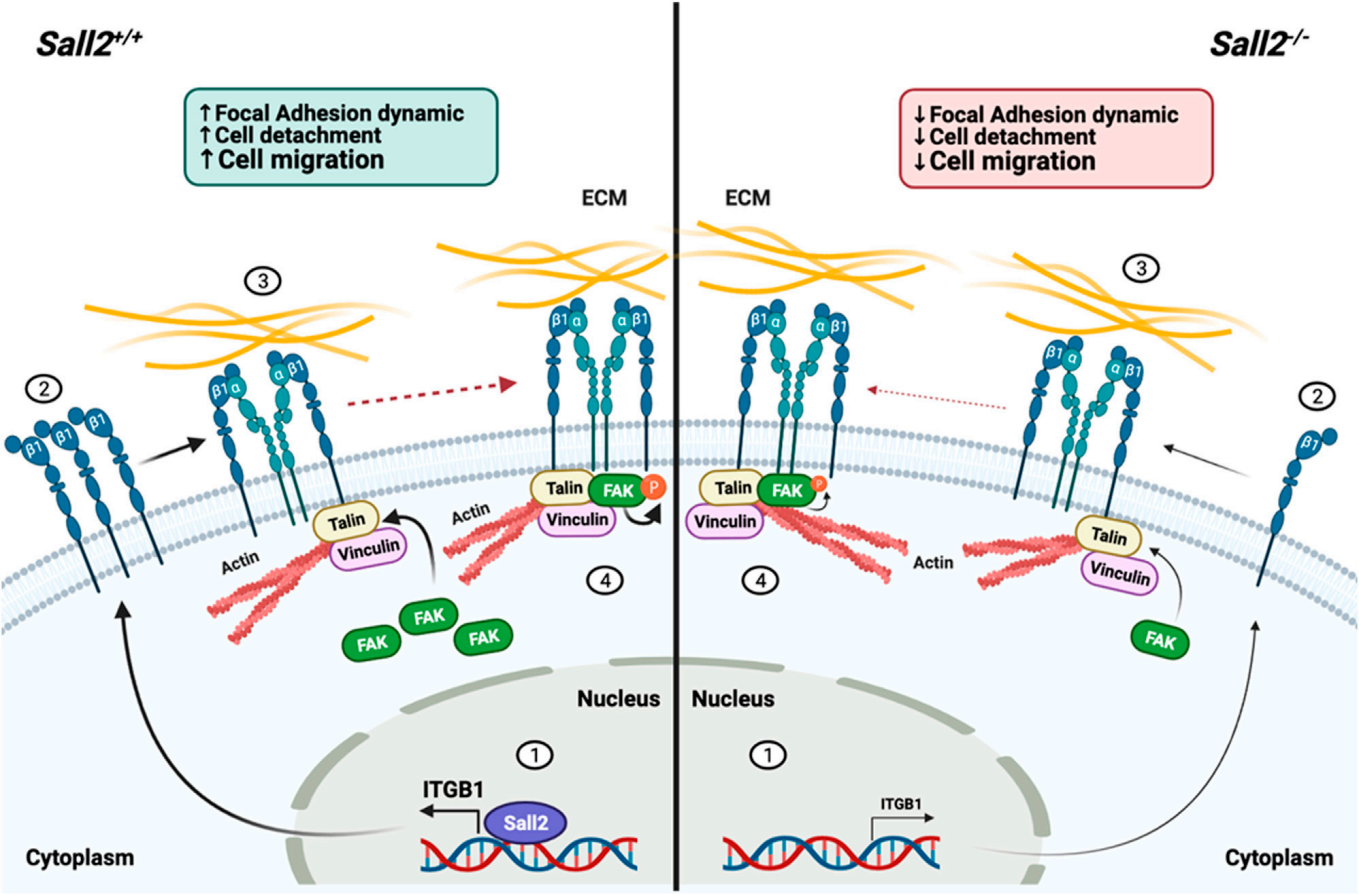
Proposed model of Sall2-dependent regulation of cell migration. In *Sall2* ^*+/+*^ iMEFs cells, **(1)** Sall2 promotes the integrin β1 mRNA expression by directly binding to and transactivating its promoter, **(2)** increasing the integrin β1 protein levels and its expression at the membrane surface, contributing in part to the integrin β1 cluster formation. **(3)** After integrin ligand-binding, actin-associated proteins like talin and vinculin bind to the integrin β1 and recruit focal adhesion kinase (FAK). **(4)** Once recruited in the adhesion site, FAK is autophosphorylated modulating FA assembly-disassembly dynamics. Conversely, in *Sall2* ^*−/−*^ cells the same chain of events occurs but less favorably. In summary, Sall2 by promoting the FA dynamics favors cell detachment and subsequently cell migration.

In summary, our results propose that Sall2 promotes cell migration by modulation of focal adhesion dynamics in a non-cancer context. This effect might relate to the direct transcriptional regulation of the *ITGB1* gene (Figure 7). Since deregulation of cell migration promotes congenital disabilities, autoimmune syndromes, and tumor formation and spreads to other tissues, our findings suggest that the Sall2-integrin β1 axis could lead to novel therapeutic approaches.

## Data Availability

The datasets presented in this study can be found in online repositories. The names of the repository/repositories and accession number(s) can be found below: https://github.com/cfarkas/SALL2_conserved_network_analysis.

## Abbreviations

ChIP, chromatin immunoprecipitation; EDTA, ethylenediaminetetraacetic acid; EMC, extracellular matrix; ESCC, esophageal squamous cell carcinoma; FA, focal adhesion; FAK, focal adhesion kinase; FN, fibronectin; iMEFs, immortalized mouse embryonic fibroblasts; KO, knockout; MTOC, microtubule-organizing center; NGF, nerve growth factor; NZ, nocodazole; NuRD, Nucleosome Remodeling Deacetylase complex; PC12, pheochromocytoma of the rat adrenal medulla; SV40, simian virus 40.
