# Supplementary material for: The Sall2 transcription factor promotes cell migration regulating focal adhesion turnover and integrin β1 expression
Source: Front Cell Dev Biol. 2022 Nov 9;10:1031262. doi: 10.3389/fcell.2022.1031262 (PMC9682130; doi:10.3389/fcell.2022.1031262)
Supplement: Supplementary file 1 [file DataSheet7.PDF]

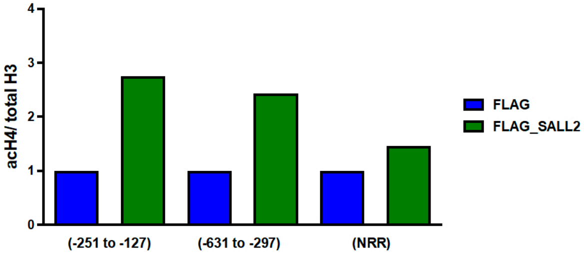

Supplementary figure 7. Changes in acetylation levels in the human integrin β1 promoter. *SALL2* KO HEK293 cells were transfected with pCDNA.3 FLAG_SALL2 vector. Chromatin was immunoprecipitated 24 h after transfection using acetylated histone H4 (AcH4) and histone H3 antibodies. Graph show changes in acetylation (AcH4) relative to total histone H3 on nucleosomes located in the −251/ −127, −631/ −297 and nonrelated (NRR) regions of *ITGB1* promoter with and without SALL2.
