# Supplementary material for: The Sall2 transcription factor promotes cell migration regulating focal adhesion turnover and integrin β1 expression
Source: Front Cell Dev Biol. 2022 Nov 9;10:1031262. doi: 10.3389/fcell.2022.1031262 (PMC9682130; doi:10.3389/fcell.2022.1031262)
Supplement: Supplementary file 2 [file DataSheet2.PDF]

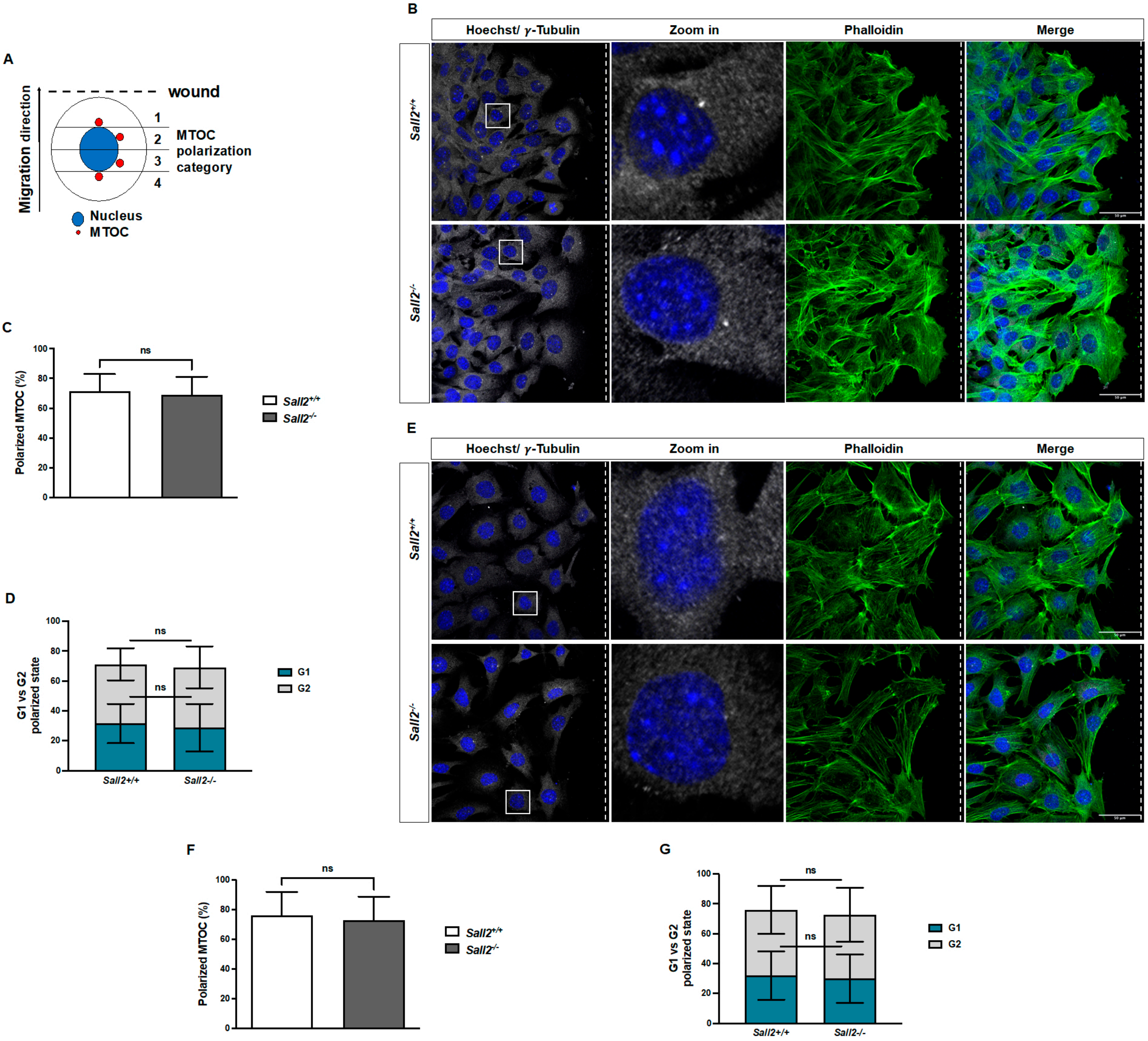

Supplementary figure 2. Sall2 does not affect cell polarity. **(A)** Schematic representation of the polarization grades used for quantifying the polarity after *in vitro* wounding. Grade 1 (G1) = highly polarized front (the MTOC was anterior to the nucleus); grade 2 (G2) = polarized front (MTOC was anterior to the midplane but behind the anterior edge of the nucleus); grade 3 (G3) = polarized rear (MTOC was posterior to the midplane but anterior to the posterior edge of the nucleus) and grade 4 (G4) = highly polarized rear (MTOC was posterior to the nucleus). **(B, E)** Representative confocal images (40x) from *Sall2*$^{+/+}$ and *Sall2*$^{-/-}$ iMEFs after cell migration induction at 4 h **(B)** and 16 h **(E)**. Samples were fixed and MTOC was immunodetected using anti-γ tubulin antibody (white), Hoechst stained the nuclei (blue) and phalloidin stained F- actin (green). Dashed lines indicate the position of the wound. **(C, F)** Percentage of polarized cells from *Sall2*$^{+/+}$ and *Sall2*$^{-/-}$ iMEFs after cell migration induction at 4 **(C)** and 16 h **(F)**. **(D, G)** Quantification of G1 and G2 polarity state from *Sall2*$^{+/+}$ and *Sall2* $^{-/-}$ iMEFs at 4 h **(D)** and 16 h **(G)**. For each experiment at least 100 cells were analyzed per genotype. Data are expressed as mean ±SD from three independent experiments (n.s, not significant; unpaired t-test).
