# Supplementary material for: The Sall2 transcription factor promotes cell migration regulating focal adhesion turnover and integrin β1 expression
Source: Front Cell Dev Biol. 2022 Nov 9;10:1031262. doi: 10.3389/fcell.2022.1031262 (PMC9682130; doi:10.3389/fcell.2022.1031262)
Supplement: Supplementary file 3 [file DataSheet4.PDF]

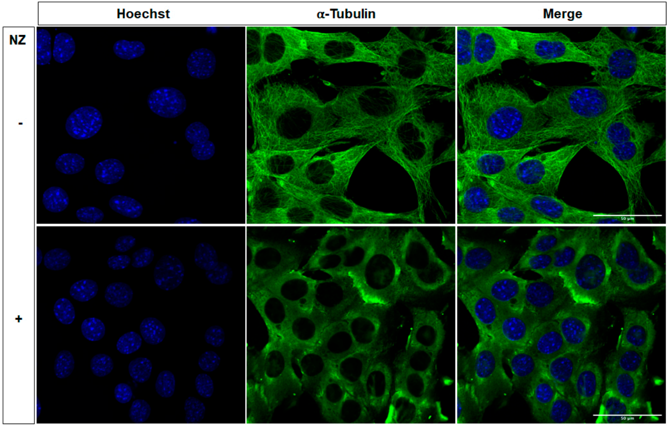

Supplementary figure 4. Microtubules depolymerization under nocodazole (NZ) synchronization. Representative confocal images (63x) from iMEFs with (+) and without (-) nocodazole treatment (10 μM, 2 ½ h). Cells were fixed, and microtubules were stained with α-tubulin antibody (green) and Hoechst stained the nuclei (blue).
