# Supplementary material for: The Sall2 transcription factor promotes cell migration regulating focal adhesion turnover and integrin β1 expression
Source: Front Cell Dev Biol. 2022 Nov 9;10:1031262. doi: 10.3389/fcell.2022.1031262 (PMC9682130; doi:10.3389/fcell.2022.1031262)
Supplement: Supplementary file 4 [file DataSheet6.PDF]

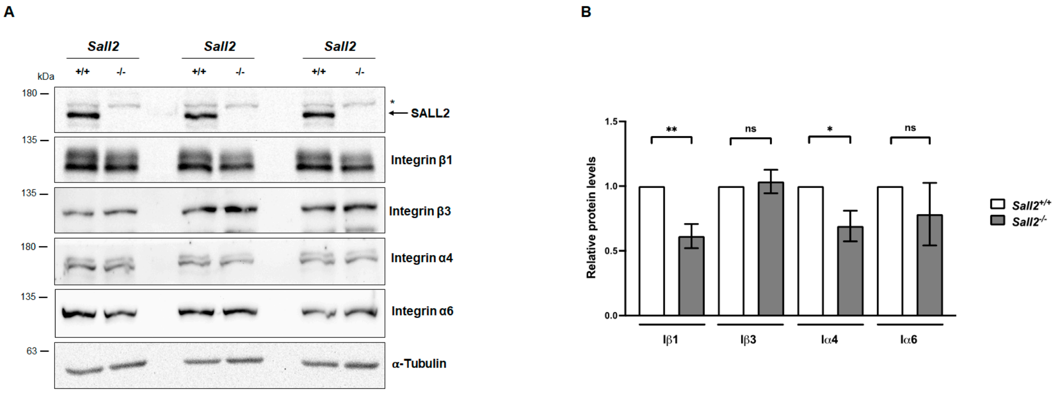

Supplementary figure 6. Sall2-dependent expression of integrins. **(A)** Representative blot of α4, α6, β1, and β3 integrin expression from *Sall2*$^{+/+}$ and *Sall2*$^{-/-}$ iMEFs growth under normal conditions. **(B)** densitometry analysis from blot obtained in A. The arrow indicates Sall2, and the asterisk corresponds to a nonspecific band. α-tubulin was used as the loading control. Data are expressed as mean ±SD from three independent experiments (n.s, not significant, *P=0.01 to 0.05, **P= 0.001 to 0.01; unpaired t-test).
