# Supplementary material for: The Sall2 transcription factor promotes cell migration regulating focal adhesion turnover and integrin β1 expression
Source: Front Cell Dev Biol. 2022 Nov 9;10:1031262. doi: 10.3389/fcell.2022.1031262 (PMC9682130; doi:10.3389/fcell.2022.1031262)
Supplement: Supplementary file 5 [file DataSheet9.PDF]

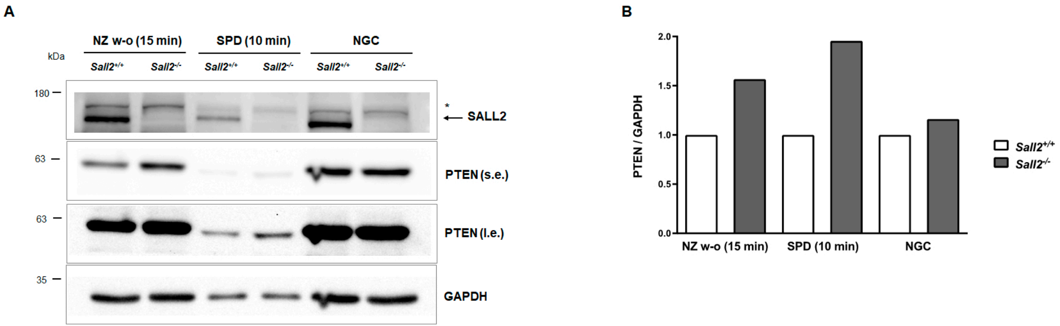

Supplementary figure 9. PTEN is downregulated in *Sall2*$^{-/-}$ iMEFs. **(A)** Blot of PTEN expression after 15 minutes of nocodazole wash-out treatment (NZ w-o), 10 minutes of spreading (SPD) on FN and under normal growth conditions (NGC) from *Sall2*$^{+/+}$ and *Sall2*$^{-/-}$ iMEFs. **(B)** Densitometric analysis of PTEN levels . The arrow indicates Sall2, and the asterisk corresponds to a nonspecific band. (s.e.) short exposure, (l.e.) long exposure. GAPDH was used as loading control.
