# Supplementary material for: The Sall2 transcription factor promotes cell migration regulating focal adhesion turnover and integrin β1 expression
Source: Front Cell Dev Biol. 2022 Nov 9;10:1031262. doi: 10.3389/fcell.2022.1031262 (PMC9682130; doi:10.3389/fcell.2022.1031262)
Supplement: Supplementary file 6 [file Table1.DOCX]

**Supplementary table 1.**  Primer sequences for qRT-PCR

| **Name** | **Sequence** | **Orientation** |
| --- | --- | --- |
| m_ITGB1_F | 5´-TGGACAATGTCACCTGGAAA-3´ | forward |
| m_ITGB1_R | 5´-TGTGCCCACTGCTGACTTAG-3´ | reverse |
| cRNA pol II_F | 5´-ACTGTGCGGAACTCCATCAA-3´ | forward |
| cRNA pol II_R | 5´-AGCCAGGTTCTGGAACTCAA-3´ | reverse |
