# Supplementary material for: The Sall2 transcription factor promotes cell migration regulating focal adhesion turnover and integrin β1 expression
Source: Front Cell Dev Biol. 2022 Nov 9;10:1031262. doi: 10.3389/fcell.2022.1031262 (PMC9682130; doi:10.3389/fcell.2022.1031262)
Supplement: Supplementary file 7 [file DataSheet3.PDF]

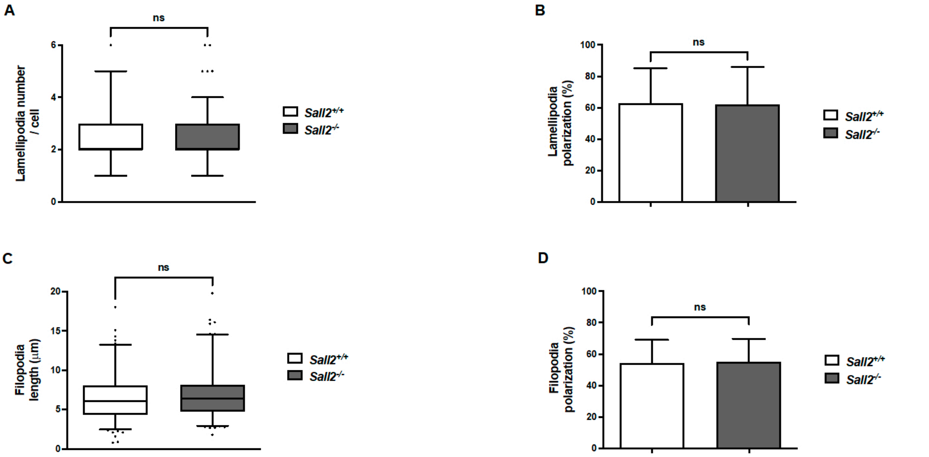

Supplementary figure 3. Sall2 role in cell membrane protrusions. **(A)** Quantification of lamellipodia number. **(C)** Quantification of filopodia length. Data of number and length are shown as a box-and-whiskers plot from three independent experiments. The whiskers represent the 2.5th and 97.5th percentile, the box extends from the 25th to the 75th percentiles and the line indicates the median. **(B, D)** Quantification of the polarization percentage of filopodia **(B)** and lamellipodia **(D)** from *Sall2*$^{+/+}$ and *Sall2*$^{-/-}$ iMEFs after cell migration induction at 16 h. Data of cell polarization are expressed as mean ±SD from three independent experiments. For each experiment at least 100 cells were analyzed per genotype. (n.s, not significant; unpaired t-test).
