# Supplementary material for: The Sall2 transcription factor promotes cell migration regulating focal adhesion turnover and integrin β1 expression
Source: Front Cell Dev Biol. 2022 Nov 9;10:1031262. doi: 10.3389/fcell.2022.1031262 (PMC9682130; doi:10.3389/fcell.2022.1031262)
Supplement: Supplementary file 8 [file Table2.DOCX]

**Supplementary table 2.**  Primer sequences for qPCR used in ChIP assay

| **Name** | **Sequence** | **Orientation** |
| --- | --- | --- |
| ChIP_hItgB1P (-251/-127)_F | 5´-CACTTCCTGCCCCATCCT-3´ | forward |
|  |  |  |
| ChIP_hItgB1P (-251/-127)_R | 5´-CTCCTCTGCGCGTCTGAT-3´ | reverse |
|  |  |  |
| ChIP_hItgB1P (-631/-297)_F | 5´-GCTTAGCCAGTTCCCTTCCA-3´ | forward |
| ChIP_hItgB1P (-631/-297)_R | 5´-GAGACAGTCGCCGTGCCTCA-3´ | reverse |
| ChIP_hItgB1P-NR (-1986/-1878)_F | 5´-TGCAGTGTGGCTTGCACTAT-3´ | forward |
| ChIP_hItgB1P-NR (-1986/-1878)_R | 5´-TGGGTGGACAGTGAGCTATG-3´ | reverse |
