# Supplementary material for: The Sall2 transcription factor promotes cell migration regulating focal adhesion turnover and integrin β1 expression
Source: Front Cell Dev Biol. 2022 Nov 9;10:1031262. doi: 10.3389/fcell.2022.1031262 (PMC9682130; doi:10.3389/fcell.2022.1031262)
Supplement: Supplementary file 9 [file DataSheet1.PDF]

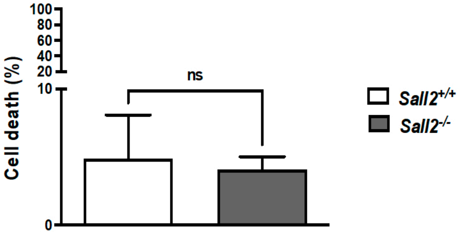

Supplementary figure 1. Sall2 does not affect cell viability under serum starvation. Quantification of *Sall2*$^{+/+}$ and *Sall2*$^{-/-}$ iMEFs viability (expressed as percentage of cell death) was evaluated at 16 h of serum starvation. Data are expressed as mean ±SD from three independent experiments performed in triplicate (n.s, not significant; unpaired t-test).
