# Supplementary material for: The Sall2 transcription factor promotes cell migration regulating focal adhesion turnover and integrin β1 expression
Source: Front Cell Dev Biol. 2022 Nov 9;10:1031262. doi: 10.3389/fcell.2022.1031262 (PMC9682130; doi:10.3389/fcell.2022.1031262)
Supplement: Supplementary file 10 [file DataSheet5.PDF]

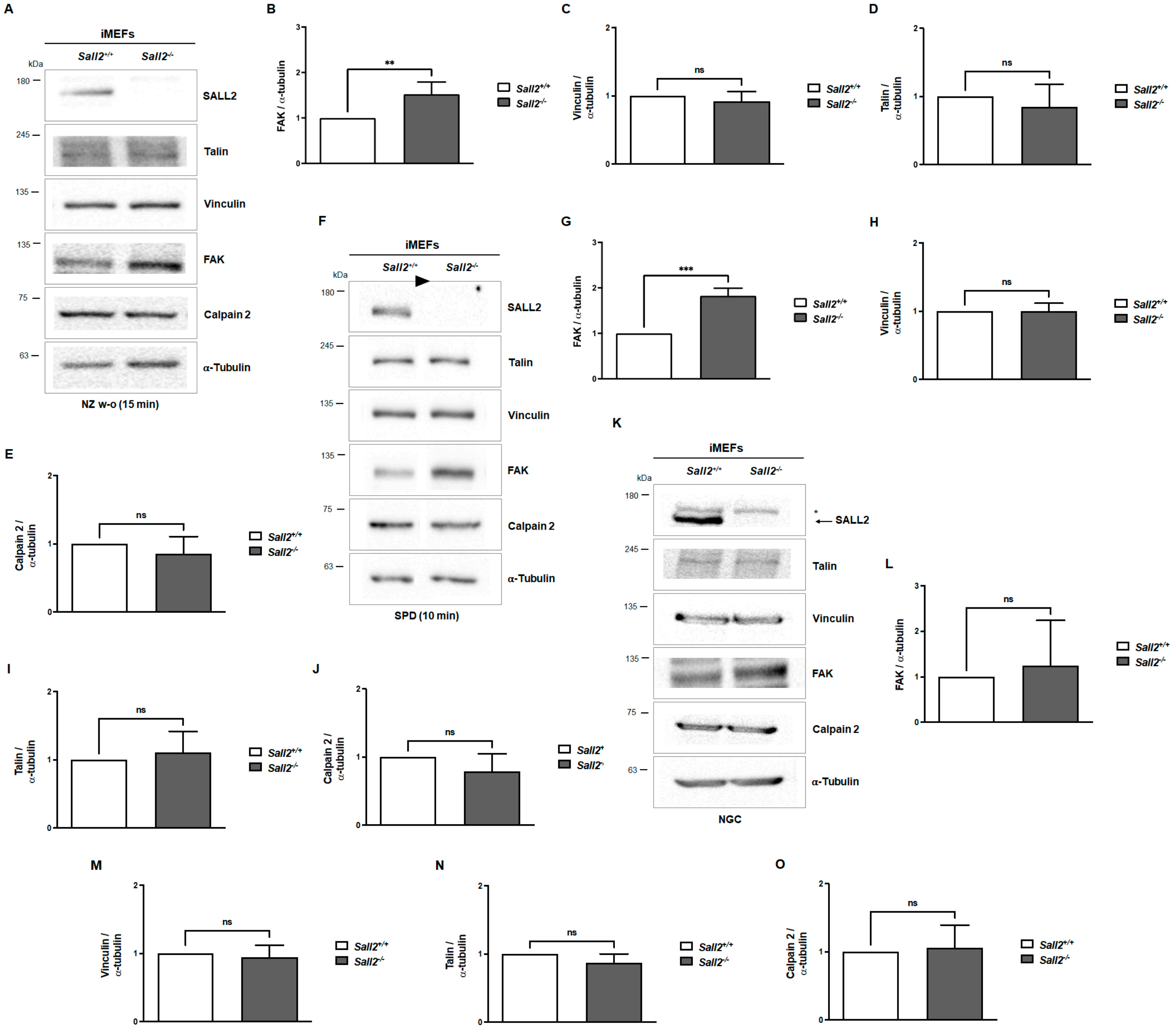

Supplementary figure 5. Sall2-dependet expression of FA assembly-disassembly proteins. **(A-E)** Representative blot **(A)**, and densitometries of FAK **(B)**, vinculin **(C)**, talin **(D)**, and calpain 2 **(E)** proteins at 15 min of nocodazole wash-out treatment (NZ w-o) from *Sall2*$^{+/+}$ and *Sall2*$^{-/-}$ iMEFs. **(F- J)** Representative blot **(F)**, and densitometries of FAK **(G)**, vinculin **(H)**, talin **(I)**, and calpain 2 **(J)** proteins after 10 min of spreading (SPD) on fibronectin (FN) from *Sall2*$^{+/+}$ and *Sall2*$^{-/-}$ iMEFs. **(K- O)** Representative blot **(K)**, and densitometries of FAK **(L)**, vinculin **(M)**, talin **(N)**, and calpain 2 **(O)** proteins under normal growth conditions (NGC) from *Sall2*$^{+/+}$ and *Sall2*$^{-/-}$ iMEFs. The arrow indicates Sall2, and the asterisk corresponds to a nonspecific band. The arrowhead in (F) indicates cropped of unrelated columns. α-tubulin was used as loading control. Data are expressed as mean ±SD from three independent experiments (n.s, not significant, $**P= 0.001$ to 0.01, $***P= 0.0001$ to 0.001; unpaired t-test).
