# Supplementary material for: The Sall2 transcription factor promotes cell migration regulating focal adhesion turnover and integrin β1 expression
Source: Front Cell Dev Biol. 2022 Nov 9;10:1031262. doi: 10.3389/fcell.2022.1031262 (PMC9682130; doi:10.3389/fcell.2022.1031262)
Supplement: Supplementary file 12 [file DataSheet8.PDF]

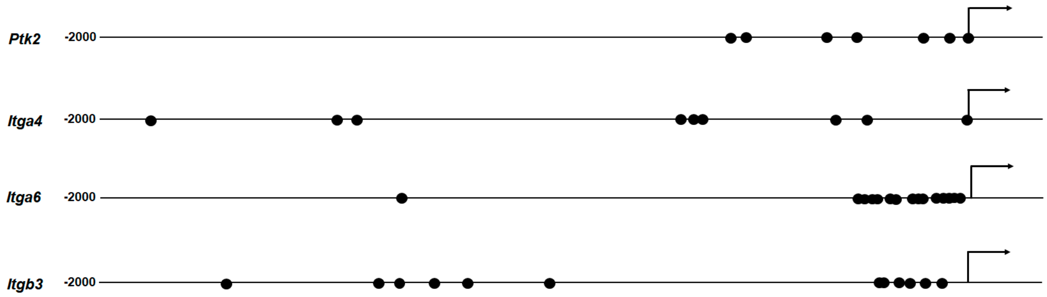

Supplementary figure 8. Schematic representation of putative Sall2 binding sites in *Ptk2*, *Itga4*, *Itga6*, and *Itgb3* mouse promoters. Analyses of gene promoters were performed in Transcriptional Regulatory Element Database (TRED). Sequences analyzed [2000 bp from transcription start site (+1)] were obtained from Eukaryotic Promoter Database (EPD). The putative Sall2 binding sites are represented by black ovals.
